# The Dark Side of the Mushroom Spring Microbial Mat: Life in the Shadow of Chlorophototrophs. II. Metabolic Functions of Abundant Community Members Predicted from Metagenomic Analyses

**DOI:** 10.3389/fmicb.2017.00943

**Published:** 2017-06-06

**Authors:** Vera Thiel, Michael Hügler, David M. Ward, Donald A. Bryant

**Affiliations:** ^1^Department of Biochemistry and Molecular Biology, The Pennsylvania State University, University ParkPA, United States; ^2^Department Microbiology and Molecular Biology, DVGW-Technologiezentrum WasserKarlsruhe, Germany; ^3^Department of Land Resources and Environmental Sciences, Montana State UniversityBozeman, MT, United States; ^4^Department of Chemistry and Biochemistry, Montana State UniversityBozeman, MT, United States

**Keywords:** hot spring, microbial community, microbial diversity, extreme environments, chlorophototrophic bacteria, metagenomics

## Abstract

Microbial mat communities in the effluent channels of Octopus and Mushroom Springs within the Lower Geyser Basin of Yellowstone National Park have been extensively characterized. Previous studies have focused on the chlorophototrophic organisms of the phyla *Cyanobacteria* and *Chloroflexi*. However, the diversity and metabolic functions of the other portion of the community in the microoxic/anoxic region of the mat are poorly understood. We recently described the diverse but extremely uneven microbial assemblage in the undermat of Mushroom Spring based on 16S rRNA amplicon sequences, which was dominated by *Roseiflexus* members, filamentous anoxygenic chlorophototrophs. In this study, we analyzed the orange-colored undermat portion of the community of Mushroom Spring mats in a genome-centric approach and discuss the metabolic potentials of the major members. Metagenome binning recovered partial genomes of all abundant community members, ranging in completeness from ~28 to 96%, and allowed affiliation of function with taxonomic identity even for representatives of novel and Candidate phyla. Less complete metagenomic bins correlated with high microdiversity. The undermat portion of the community was found to be a mixture of phototrophic and chemotrophic organisms, which use bicarbonate as well as organic carbon sources derived from different cell components and fermentation products. The presence of rhodopsin genes in many taxa strengthens the hypothesis that light energy is of major importance. Evidence for the usage of all four bacterial carbon fixation pathways was found in the metagenome. Nitrogen fixation appears to be limited to *Synechococcus* spp. in the upper mat layer and *Thermodesulfovibrio* sp. in the undermat, and nitrate/nitrite metabolism was limited. A closed sulfur cycle is indicated by biological sulfate reduction combined with the presence of genes for sulfide oxidation mainly in phototrophs. Finally, a variety of undermat microorganisms have genes for hydrogen production and consumption, which leads to the observed diel hydrogen concentration patterns.

## Introduction

Microbial mat communities inhabiting the effluent channels of Octopus and Mushroom Springs within the Lower Geyser Basin at Yellowstone National Park (YNP) have been studied for nearly 50 years (Brock, 1967; Ward et al., 2012). In these studies, the chlorophototrophic bacterial populations, i.e., chlorophyll (Chl)-based phototrophs including members of the *Cyanobacteria, Chloroflexi*, and the recently discovered *Chloracidobacterium (Cab.) thermophilum* and “*Candidatus* Thermochlorobacter aerophilum,” have generally been the main focus (Bauld and Brock, 1973; Nold and Ward, 1996; Bryant et al., 2007; van der Meer et al., 2007; Steunou et al., 2008; Becraft et al., 2011; Klatt et al., 2011, 2013b; Liu et al., 2011, 2012; Tank and Bryant, 2015a,b; Tank et al., 2017). Only recently, the diversity of the microbial community in the microoxic/anoxic region of the mat has been explored (Thiel et al., 2016).

**Figure 1:**
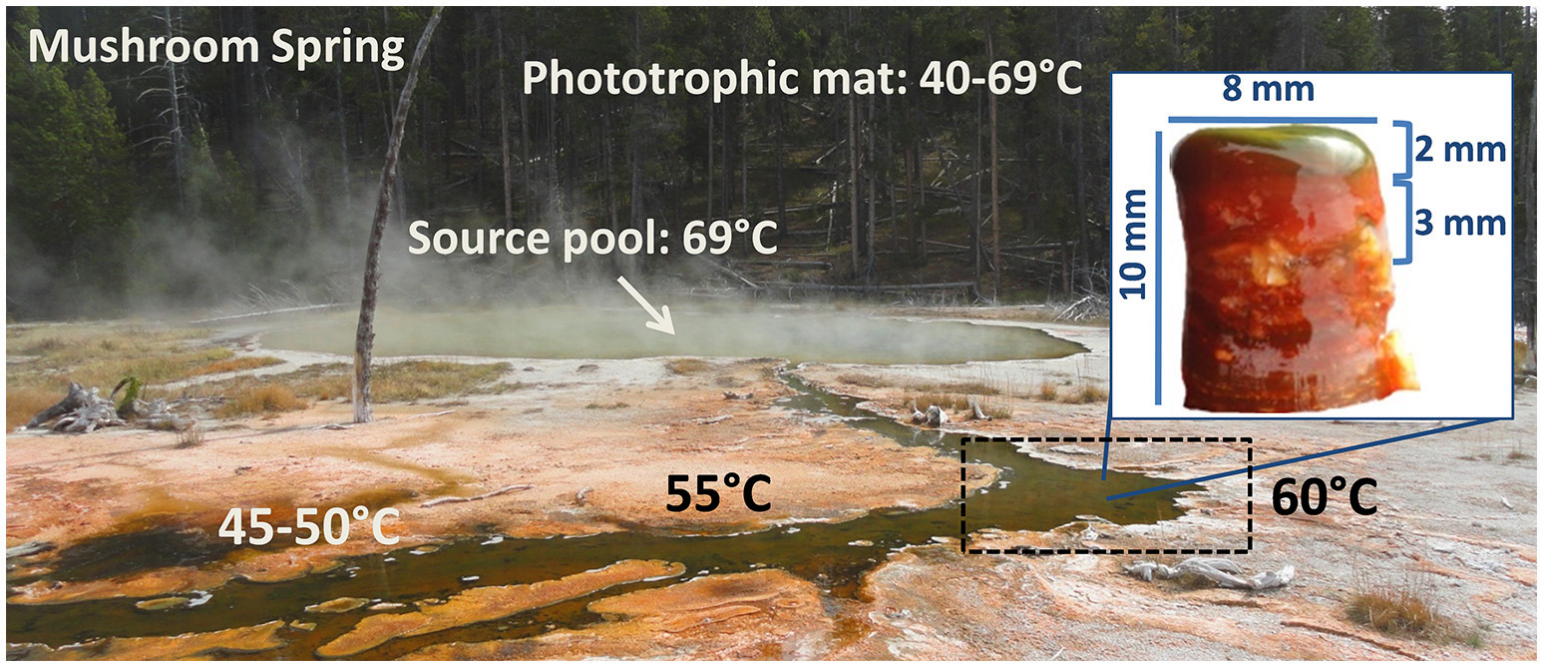
**Picture of Mushroom Spring in Yellowstone National Park and a microbial mat core, taken at 60°C**.

The upper green layer of the mats of Mushroom Spring and/or Octopus Spring (Figure 1) have been studied using metagenomic (Klatt et al., 2011), metatranscriptomic (Liu et al., 2011, 2012; Klatt et al., 2013b), metaproteomic (Schaffert et al., 2012), and metametabolomic (Kim et al., 2015) analyses. This layer is dominated by six chlorophototrophic bacterial taxa, in particular two types of cyanobacteria, *Synechococcus* sp. Types A and B' (Bhaya et al., 2007), chlorophototrophic members of the *Chloroflexi* (*Roseiflexus* sp., *Chloroflexus* sp., and “Ca. Roseilinea gracile”; Klatt et al., 2011, 2013b; Tank et al., 2017) as well as two unusual microaerophilic anoxygenic photoheterotrophs, *Chloracidobacterium thermophilum* (Bryant et al., 2007; Garcia Costas et al., 2012a,b; Tank and Bryant, 2015a,b) and “*Ca*. Thermochlorobacter aerophilum” (Liu et al., 2012; Tank et al., 2017). Additionally, two heterotrophic taxa were found in a metagenomic analysis (Klatt et al., 2011). They were later identified as members of the phylum *Armatimonadetes* and the EM3 group tentatively affiliated with the *Thermotogae* (Thiel et al., 2016).

The microbial composition of the orange-colored undermat was assessed in a previous study using metagenomic sequencing and amplicon studies of 16S rRNA genes (iTag) (Thiel et al., 2016). In comparison to the upper green layer, the Mushroom Spring undermat part of the community was shown to be highly diverse but very uneven; it is dominated by members of a single genus, *Roseiflexus*, that includes filamentous anoxygenic phototrophs, which also inhabit the upper green layer as shown by molecular- and microscopy-based studies (“OS type C”; Nübel et al., 2002; Ward and Cohan, 2005; Klatt et al., 2007, 2013a,b; Thiel et al., 2016; Tank et al., 2017). In addition to the members of the upper green layer of the mat, all of which were also detected in the undermat in lower abundance, further photo- and chemotrophic bacteria were identified. *Pseudothermotoga* sp. was the second most abundant member. Furthermore, a novel *Armatimonadetes*, a member of the *Aquificae* (*Thermocrinis* sp.) and a novel chlorophototrophic member of the phylum *Chloroflexi*, “*Ca*. Roseilinea gracile,” were identified as abundant members of the undermat (Thiel et al., 2016). Less abundant taxa, but still representing ≥1% of the undermat part of the microbial community based on amplicon reads, were a member of the phylum *Atribacteria* (OP9/JS1); a sulfate-reducing *Thermodesulfovibrio* sp.; a member of the phylum *Planctomycetes*; a member of the EM3 group tentatively affiliated with the *Thermotogae*, as well as a putative member of the *Arminicenantes* (OP8). According to the iTag analysis, *Archaea* are rare, and no metagenomic bin representing an archaeon was identified. At present, little is known about the physiological, metabolic, and functional potentials of these heterotrophic members of the undermat layers of the community.

The overall goal of this research is to investigate the complete microbial mat community at Mushroom Spring and to develop a comprehensive understanding of the microbial ecology of the microbial mats of this hot spring. As noted above, the composition and diversity of the undermat was described in a previous report (Thiel et al., 2016). In this report, we focus on a description of the metabolic potentials and hypothesized interactions among mat community members.

## Materials and methods

### Sampling, DNA extraction, metagenomic sequencing, and binning

Sample collection, DNA extraction, and DNA sequencing were performed as described (Thiel et al., 2016). In brief, the samples were collected on August 10th, 2011 from a microbial mat in an effluent channel of the siliceous and slightly alkaline Mushroom Spring in the Lower Geyser Basin of YNP, WY (USA). Samples were collected using a #4 cork borer (⊘ = 9 mm) at a site where the water above the mat was 60°C (Figure 1). The microbial mat is made up of an upper green layer (1–2 mm thick), which mainly consists of different chlorophototrophic bacteria, and an orange-colored undermat layer. Genomic DNA was extracted from the orange-colored undermat layer from a depth of ~3–5 mm; tests showed that DNA from below this level was too degraded to analyze. The metagenomic DNA was sequenced at the DOE Joint Genome Institute (JGI) using HiSeq Illumina technologies. HiSeq DNA sequences were assembled and then clustered into bins by oligonucleotide frequency pattern analyses using the emergent self-organizing map (ESOM) method described by Dick et al. (2009). Initial binning was conducted with sequences ≥5.0 kb in length (Thiel et al., 2016), whereas in this study binning was conducted using sequences ≥2.5 kb. Reference genomes as well as metagenomic bin information from the binning of ≥5.0 kb sequences was used for initial identification of the bin. Metagenomic bins were treated as partial genomes of single taxa, although discussion of ecotype diversity within these taxa is raised where appropriate below; Amphoranet (http://pitgroup.org/amphoranet/; Kerepesi et al., 2014) was used to assess the phylogenetic marker genes present in each bin and to assign taxonomic affiliations. Completeness of the partial genomes was further assessed using CheckM (Parks et al., 2015). The partial genomes and reference genomes of closely related bacteria were annotated using RAST and used to assess and compare the physiological and metabolic potential of mat members (Table 1; Aziz et al., 2008; Overbeek et al., 2014). Detailed descriptions of the methods for DNA extraction, library construction, sequencing, and data analyses are found in the Supplementary Materials in Thiel et al. (2016).

**Table 1:** **List of metagenomic bins and reference genomes used in this study**.

**Bin/OTU**	**Identity**	**16S rRNA**	**16S rRNA phylogeny**	**Contigs**	**Size (Mb)**	**N50 (bp), contig #**	**Average contig length (kb)**	**max contig length (kb)**	**GC%**	**Average coverage**	**Completeness (checkM) (%)**	**Contamination (checkM)^&^**	**Number (and average copy) of Amphora marker genes^$^**	**Amphora taxonomy (% of genes supporting)**	**ANI^+^ (Coverage)**	**Next relative^+^**	**Metagenome bin RAST ID**	**Reference genome RAST ID genome size GC%**
1	*Chloroflexi, Roseiflexus* sp., OS type C	No	n.a.	312	1.10	3,384, *n* = 117	3.61	25.05	60^a^	787x^a^	28	0.00	20 (1.0)	*Bacteria; Chloroflexi; Chloroflexi; Chloroflexales; Chloroflexaceae; Roseiflexus* (100)	96.27 (75.44)	*Roseiflexus* sp. RS-1	6666666.252793	*Roseiflexus* sp. RS-1 357808.8 5.80 Mb 60%
2	*Thermotogae, Pseudohermotoga* sp.	Yes	*Thermotogae - Thermotogales - Pseudothermotoga*	203	2.01	13,293, *n* = 45	10.17	53.33	50	421x	93	0.16	31 (1.0)	*Bacteria; Thermotogae; Thermotogae; Thermotogales; Thermotogaceae; Thermotoga* (100)	87.64% (91.71%)	*Pseudothermotoga hypogea* DSM 11164	6666666.236741	*Pseudothermotoga hypogea* DSM 11164 1123384.6 2.16 Mb 50%
3^b^	*Armatimonadetes* (OP10), Group 7, OS type L	Yes	*Armatimonadetes -* group 7	81	2.95	57,852, *n* = 18	37.29	142.7	63	n.d.	96	0.00	31 (1.0)	*Bacteria; Armatimonadetes* (100; BLASTp)	73.44 (47.64)	*Armatimonadetes* bacterium DC	6666666.122993	n.a.
4	*Aquificae, Thermocrinis* sp.	Yes	*Aquificae - Aquificaceae - Thermocrinis*	291	1.12	3,859, *n* = 106	3.93	12.07	45	161x	43	22.14	9 (1.0)	*Bacteria; Aquificae; Aquificae; Aquificales; Aquificaceae* (100)	92.56 (85.60)	*Thermocrinis ruber* DSM 23557	6666666.252516	*Thermocrinis ruber* DSM 12173 932678.4 1.52 Mb 45%
5	*Cyanobacteria, Synechococcus* sp. B', OS type B'	No	n.a.	357	1.42	4,241, *n* = 132	4.06	13.01	59	71x	53	0.88	19 (1.0)	*Bacteria; Cyanobacteria; Chroococcales; Synechococcus; Synechococcus* sp. JA-2-3B'a (2–13) (100)	97.93 (93.96)	*Synechococcus* sp. JA-2-3Ba 2-13 JA-2-3B'a(2-13)	6666666.252508	*Synechococcus* sp. JA-2-3B'a (2-13) 321330.3 3.05 Mb 45%
6	*Chloroflexi*, “*Ca*. Roseilinea gracile”	Yes	*Chloroflexi -* uncultured	440	2.63	7,110, *n* = 117	6	37.32	63	320x	78	2.73	27 (1.0)	*Bacteria* (100); *Chloroflexi* (96); *Anaerolineaceae* (89)	no relevant hits^#^	no relevant hits^#^	6666666.201053	n.a.
7	*Atribacteria* (OP9)	No	n.a.	136	1.44	11,114, *n* = 40	10.57	42.24	51	104x	58	0.15	26 (1.04)	*Bacteria* (100)	68.45 (51.03)	“*Candidatus* Caldatribacterium saccharofermentans”	6666666.193399	n.a.
8	*Nitrospirae, Thermodesulfovibrio* sp.	Yes	*Nitrospirae - Nitrospiraceae - Thermodesulfovibrio*	208	1.93	14,141, *n* = 41	9.3	40.03	33	109x	96	0.00	31 (1.0)	*Bacteria; Nitrospirae; Nitrospira; Nitrospirales; Nitrospiraceae; Thermodesulfovibrio; T. yellowstonii* (100)	76.40 (61.68)	*Thermodesulfovibrio yellowstonii* DSM 11347	6666666.208104	*Thermodesulfovibrio yellowstonii* DSM 11347 289376.12 2.00 Mb 34%
9	*Chloroflexi, Anaerolineae*	Yes	*Chloroflexi-Anaerolineae*	354	3.01	11,510, *n* = 70	8.51	54	52	118x	82	4.55	30 (1.0)	*Bacteria; Chloroflexi* (100) *Anaerolineae; Anaerolineales; Anaerolineaceae; Anaerolinea* (97)	65.80 (20.33)	*Anaerolinea thermophila* UNI-1 (TCS: 0.087221) *Thermanaerothrix daxensis* GNS-1	6666666.207742	n.a.
10	*Thermotogae*-EM3	Yes	*Thermotogae*-EM3	337	2.00	6,639, *n* = 95	5.95	23.47	57	72x	82	2.93	29 (1.0)	*Bacteria* (100)	no relevant hits^#^	no relevant hits^#^	6666666.208901	n.a.
11	*Chloroflexi, Chloroflexus* sp.	Yes	*Chloroflexi-Chloroflexaceae-Chloroflexus*	720	4.5	7,532, *n* = 196	6.32	33.81	54	45x	94	11.32	30 (1.0)	*Bacteria; Chloroflexi; Chloroflexi; Chloroflexales; Chloroflexaceae; Chloroflexus* (97)	97.63% (89.36%)	*Chloroflexus* sp. MS-G	6666666.209729	*Chloroflexus* sp. MS-G 1107.5 4.77 Mb 54%
12	*Armatimonadetes (OP10)*, Group 6	Yes	*Armatimonadetes*- group 6	466	2.64	6,435, *n* = 130	5.66	25.71	60	405x	73	1.95	27 (1.0)	*Bacteria* (100); *Armatimonadetes* (67; BLASTp)	66.53 (16.15)	*Armatimonadetes* bacterium GBS	6666666.236755	n.a.
13	*Aminicenantes* (OP8)	No	n.a.	95	2.52	48,700, *n* = 20	26.81	92.13	47	42x	92	7.69	30 (1.0)	*Bacteria* (100); *Aminicenantes* (97; BLASTp)	64.10 (21.67)	*Aminicenantes* bacterium SCGC AAA252-A02	6666666.139726	n.a.
14	*Planctomycetes*	Yes	*Planctomycetes*- Pir3 lineage	792	3.73	4,907, *n* = 259	4.72	15.26	61	62x	78	10.53	31 (1.0)	*Bacteria; Planctomycetes; Planctomycetia; Planctomycetales; Planctomycetaceae* (100)	no relevant hits^#^	*Singulisphaera acidiphila* DSM 18658 (TCS: 0.6372)	6666666.208066	n.a.
15	*Chloroflexi*, “*Ca*. Chloranaerofilum corporosum”	No	n.a.	817	4.18	5,455, *n* = 252	5.1	38.26	67	49x	64	2.23	20 (1.0)	*Bacteria; Chloroflexi; Chloroflexia* (100); *Chloroflexales; Chloroflexaceae* (90)	70.45 (29.28)	*Oscillochloris trichoides* DG-6	6666666.236756	n.a.

a Averages for coverage and GC content of OTU-1 was determined from scaffolds with genus affiliation Roseiflexus as determined by IMG/MER;

b OTU-3 partial; genome was obtained from an enrichment culture metagenome;

$ duplicate marker genes with (an overlap with) >95% aa identity were considered as single gene copy;

# genome comparisons were only listed when ≥20% nucleotides were matched with ≥60% identity;

+ next relative genome and average nucleotide identity (ANI) values determined by blastn or tetranucleotide frequency (TCS; if stated) using JSpeciesWS; n.a., not applicable;

& *CheckM contamination values are percentages. Contamination >100% indicates the recovered bin likely contains multiple organisms (https://github.com/Ecogenomics/CheckM/issues/65)*.

### Metagenomic analysis

In addition to the partial genomes, the assembled metagenome was analyzed separately. For functional analyses of the metagenome, the “Gene Search” and “BLAST” functions implemented in the JGI Integrated Microbial Genomes and Microbiome Samples Expert Review (IMG/MER) system (https://img.jgi.doe.gov/cgi-bin/mer/main.cgi) were used. Amino acid sequences of predicted open reading frames were subjected to searches against the NCBI conserved domain database (CDD; http://www.ncbi.nlm.nih.gov/Structure/cdd/cdd.shtml; Marchler-Bauer and Bryant, 2004; Marchler-Bauer et al., 2009, 2011, 2015) as well as to BLASTp searches against the “non-redundant sequences” (nr) database (http://blast.ncbi.nlm.nih.gov/Blast.cgi; Altschul et al., 1990, 1997) to verify function and taxonomic affiliation.

### Phylogenetic analyses

Ribosomal RNA genes were extracted and downloaded from the annotated JGI metagenome dataset ID 3300002493. Phylogenetic analyses of ribosomal RNA sequences, as well as functional genes, were conducted using ARB software for sequence analysis (Ludwig et al., 2004). Publicly available databases for 16S rRNA and *dsrAB*/DsrAB sequences (SILVA SSU Ref NR 123 database released in July 2015: http://www.arb-silva.de/projects/ssu-ref-nr/; and dsrab_dome_v3.arb, http://www.microbial-ecology.net/download; Müller et al., 2015) were used. Databases for additional functional genes were specifically created *de novo*. Sequences were imported as amino acid or nucleotide sequences in FASTA format and aligned using the ClustalW Protein Aligner (slow and accurate settings) implemented in the ARB software package; the alignments were then inspected and edited manually. Using the PHYML software implemented in the ARB package, phylogenetic trees were constructed with the Maximum Likelihood method; the inferred confidence was based on 100 bootstrap replicates.

### Taxonomic identification

Affiliation to specific mat members was inferred by nucleotide frequency-based binning using ESOM if gene sequences were located on scaffolds with nucleotide lengths of >2.5 kb. Previously observed bins obtained with >5.0 kb length sequences (Thiel et al., 2016), as well as included reference genomes (Table S1) and phylogenetic marker genes determined by Amphoranet (http://pitgroup.org/amphoranet/; Kerepesi et al., 2014), were further used to identify bins. Because Amphoranet uses an outdated NCBI taxonomy file version (last updated 2011; https://www.biostars.org/p/122761/; C. Kerepesi, pers. commun.), additional BLASTp searches were conducted for marker gene sequences that produced ambiguous results and/or were obtained from recently described or candidate phyla. Additional information on the taxonomic identity of scaffolds and genes was obtained from the “phyloDist” method implemented in the JGI IMG/MER software as well as from BLAST search results. All data used in this study is available through public databases. The metagenome is available at IMG/M and IMG/MER under genome ID 330002493. RAST annotation jobs of all (partial) genomes used in this study are publically available using the guest account for the RAST online database (http://rast.nmpdr.org/; username “guest” with password “guest”; for job ID information see Table 1). The JSpecies Web Server (JSpeciesWS; Richter et al., 2015) was used to determine most closely related genomes by tetranucleotide search (TCS) and average nucleotide identity (ANI) values.

## Results and discussion

### Introductory comments

In this study, we analyzed the 232-Mb assembled metagenome of the orange-colored undermat of the chlorophototrophic microbial mat at Mushroom Spring in Yellowstone National Park. The focus of this analysis was the metabolic lifestyle and genetic potentials of the 15 most abundant mat members identified by 16S rRNA gene amplicon study (Thiel et al., 2016), as well as nutrient cycling within the mat community. Tetranucleotide frequency patterns were used to bin the metagenomic sequences in order to identify partial genomes of uncultured mat members. In an initial approach, binning of 5,362 contigs with length of >5 kb resulted in 37 partial genomes (Thiel et al., 2016). In this study we conducted another binning analysis that included scaffolds with lengths >2.5 kb (13,766 contigs). The resulting metagenomic bins were largely congruent with the previously defined bins (Thiel et al., 2016) and were identified by these congruencies in combination with phylogenetic marker gene analyses and additional reference genomes now included in the binning process (Table S1). The newly obtained metagenomic bins representing the most abundant members of the undermat, as indicated by a 16S rRNA amplicon study (Thiel et al., 2016), are shown in Table 1.

The partial genomes as well as the entire metagenome were independently analyzed to identify genes encoding key enzymes of different metabolic pathways. Results discussed in the following sections are mainly based on genes present in sequence bins. Due to the incomplete character of the genomes, inferences concerning metabolism can only be deduced from the presence but not the absence of genes for diagnostic enzymes. Genes for key enzymes that were present in the assembled but unbinned metagenome were also identified; this analysis was performed to identify specific genes present on short scaffolds that were excluded in the binning approach. When unbinned genes from the metagenome could unambiguously be affiliated with a specific community member, they were included in the metabolic predictions for that organism. In particular, genes encoding key enzymes in phototrophic and chemotrophic energy production, as well as nutrient (carbon, nitrogen, and sulfur) metabolism were analyzed.

The whole mat community was found to be a mixture of photo- and chemo-trophic organisms, using bicarbonate as well as organic carbon sources derived from different cell components and fermentation products. The mat community, which was originally described as a “algal/cyanobacterial mat,” contained a panoply of chlorophototrophs (Doemel and Brock, 1976; Brock, 1995; Bryant et al., 2007; Klatt et al., 2011; Thiel et al., 2016; Tank et al., 2017). Based on 16S rRNA sequence analysis, most of the chlorophototrophs were higher in abundance in the upper green layer, while one abundant as well as two low-abundance chlorophototrophs were detected almost entirely in the undermat (Thiel et al., 2016).

The surprising finding of diverse rhodopsin genes (see below) strengthens the conclusion that light energy is of paramount importance in these mats. Carbon fixation pathways are similarly diverse, and representatives of the *Bacteria* performing carbon fixation by each of the four known autotrophic pathways in *Bacteria* were identified in the metagenome. Cyanobacterial nitrogen fixation appears to be the main source for nitrogen, which is made available to the mat community in forms of amino acids and ammonium, while dissimilatory nitrogen metabolism was not detected. A closed sulfur cycle is hypothesized to be maintained by biological sulfate reduction in combination with sulfide:quinone reductase (SQR)-based sulfide oxidation and sulfur oxidation (SOX) system-mediated sulfur, sulfite, and thiosulfate oxidation (Friedrich et al., 2001; Dahl and Friedrich, 2008; Härtig et al., 2014). Sulfur disproportionation is predicted to occur in one low-abundance mat member. Finally, hydrogen is produced and consumed in the mat community by a variety of organisms leading to diel concentration patterns, which have been measured using microsensors (Revsbech et al., 2016).

### Light utilization: chlorophototrophy and retinalophototrophy

#### Chlorophototrophy

Light is the most important energy source for the phototrophic microbial mat at Mushroom Spring. Although it has often been referred to as a “cyanobacterial mat” (Doemel and Brock, 1976; Brock, 1995; Ward et al., 1998; Ramsing et al., 2000; Klatt et al., 2011) highly diverse phototrophic bacteria inhabit the mat community. Sixteen different chlorophototrophic bacteria have been identified by a combination of molecular and isolation- and enrichment-based studies (Table 2; Klatt et al., 2011; Thiel et al., 2016; see Figure 4 in Tank et al., 2017). Genes encoding type-1 photosynthetic reaction center (RCs; *psaA/psaB* and *pscA*) and type-2 RCs (*pufLM, psbA*, and, *psbD*) of 10 chlorophototrophs were identified in the undermat metagenome at 60°C (Table S2). Taxa containing type-2 RCs are the dominant oxygenic chlorophototrophic *Cyanobacteria*: *Synechococcus* spp. Types A and B' (OTUs 22 and 5, respectively); members of the *Chloroflexi*: *Roseiflexus* spp. (OTU-1), “*Ca*. Roseilinea gracile” (OTU-6), *Chloroflexus* sp. (OTU-11) and “*Ca*. Chloranaerofilum corporosum” (OTU-15); as well as two less abundant chlorophototrophic *Alphaproteobacteria*: “*Ca*. Elioraea thermophila” (OTU-46) and “*Ca*. Roseovibrio tepidum” (OTU-121; Table S2; Thiel et al., 2016; Tank et al., 2017).

**Table 2:** **Microbial mat members containing genes for chlorophototrophy and/or rhodopsin**.

**Taxa**	**Chloro-phototroph**	**Reaction center type**	**Rhodopsin**	**OTU ^$^**	**Rel. abundance undermat^$^ (%)**	**Rel. abundance upper layer^$^ (%)**	**Culture**	**Literature**
*Roseiflexus* sp. RS-1	Yes	Type-2	Yes	OTU-1	49.07	33.93	Isolate	van der Meer et al., 2010
*Armatimonadetes* member	No	n.a.	Yes	OTU-3	4.45	0.83	Uncultured	Thiel et al., 2016
*Synechococcus* sp. B'	Yes	PS1, PS2	No	OTU-5	3.62	37.36	Isolate	Bhaya et al., 2007
“*Ca*. Roseilinea gracile”	Yes	Type-2	Yes	OTU-6	2.57	1.21	Enrichment	Klatt et al., 2011; Thiel et al., 2016; Tank et al., 2017
*Chloroflexus* sp. MS-G	Yes	Type-2	No	OTU-11	1.14	1.24	Isolate	Thiel et al., 2014
“*Ca*. Chloranaerofilum corporosum”	Yes	Type-2	No	OTU-15	0.72	0.00	Enrichment	Thiel et al., 2016; Tank et al., 2017
*Chloracidobacterium thermophilum*	Yes	Type-1	No	OTU-17	0.65	5.20	Isolate	Bryant et al., 2007; Tank and Bryant, 2015a,b
*Meiothermus* sp.	No	n.a.	Yes	OTU-21	0.47	0.44	Isolate	Thiel et al., 2015
*Synechococcus* sp. A	Yes	PS1, PS2	No	OTU-22	0.46	0.48	Isolate	Bhaya et al., 2007; Nowack et al., 2015; Olsen et al., 2015
*Bellilinea* sp.	No	n.a.	Yes	OTU-31	0.34	0.07	Uncultured	Thiel et al., 2016
“*Ca*. Thermochlorobacter aerophilum”	Yes	Type-1	No	OTU-38	0.18	2.24	Enrichment	Klatt et al., 2011; Liu et al., 2012; Tank et al., 2017
“*Ca*. Elioraea thermophila”	Yes	Type-2	No	OTU-46	0.09	0.01	Enrichment	Tank et al., 2017
*Thermus* sp.	No	n.a.	Yes	OTU-70	0.04	0.00	Uncultured	Thiel et al., 2016
*Isosphaera-*like *Planctomycetes*	No	n.a.	Yes	OTU-83	0.02	0.00	Uncultured	Thiel et al., 2016
“*Ca*. Roseovibrio tepidum”	Yes	Type-2	No	OTU-121	0.01	0.00	Enrichment	Tank et al., 2017
*Thermochromatium tepidum*	Yes	Type-2	No	OTU-226	0.00	0.00	Isolate	Tank et al., 2017
*Synechococcus* sp. C9	Yes	PS1, PS2	No	OTU-279	0.00	0.00	Isolate	Ferris et al., 1996
*Thermosynechococcus* sp. C1	Yes	PS1, PS2	No	n.d.	n.d.	n.d.	Isolate	Ferris et al., 1996
OS type I (*Leptolyngbya* sp.)	Yes	PS1, PS2	No	n.d.	n.d.	n.d.	Uncultured	Ward et al., 1992; Weller et al., 1992
OS type J (*Synechococcus* sp.)	Yes	PS1, PS2	No	n.d.	n.d.	n.d.	Uncultured	Ward et al., 1992; Weller et al., 1992
*Blastochloris* sp.	Yes	Type-2	No	n.d.	n.d.	n.d.	Isolate	Tank et al., 2017
*Heliobacterium modesticaldum*	Yes	Type-1	No	n.d.	n.d.	n.d.	Isolate	Kimble et al., 1995

n.a., Not applicable; n.d., not determined/unknown;

$ *based on 16S rRNA gene amplicon analysis (Thiel et al., 2016)*.

Aside from *Synechococcus* spp. Types A and B' (OTUs 22 and 5, respectively), taxa containing Type-1 RCs, *Chloracidobacterium thermophilum* (Bryant et al., 2007; Garcia Costas et al., 2012b; Tank and Bryant, 2015a,b) and “*Ca*. Thermochlorobacter aerophilum” (Liu et al., 2012), which were first identified in the upper layer of the mat, were also detected in the undermat metagenome (Table S2). Although *Heliobacterium modesticaldum* was previously isolated from these same mats in a region of lower temperature (Kimble et al., 1995), no *pshA* sequences, encoding for the P800 photosynthetic reaction center core protein of heliobacteria, were identified nor did the reference genome of this organism recruit any significant hits from the metagenome. This is presumably because *H. modesticaldum* does not grow at temperatures above ~56°C (Kimble et al., 1995). Similarly, no *psaA* and *psaB* genes affiliated with *Thermosynechococcus* sp. C1, *Synechococcus* sp. C9, or *Leptolyngbya* sp. were identified in the metagenome, although these organisms have previously been isolated from these mats (Tank et al., 2017 and references therein). This is probably explained either by their very low abundance and/or optimal growth temperatures lower than that studied here. Similarly, *Thermochromatium tepidum* and *Blastochloris* sp. have been obtained through cultivation studies from lower temperature mat samples (~50°C; Tank et al., 2017), but were not detected in the 60°C undermat metagenome.

The highest diversity in genes for type-2 reaction centers was seen for members of OTU-1, *Roseiflexus* spp. (16 partial *pufLM* genes with coverage values ranging from 71 to 1227 × Table S2). This indicates that several *Roseiflexus* spp. ecotypes with differences in their RC proteins are likely present in the mat community. Similarly, cyanobacterial scaffolds containing RC genes showed high diversity, suggesting the presence of different ecotypes adapted to specific light niches as shown previously (Table S2; Becraft et al., 2015; Nowack et al., 2015; Olsen et al., 2015). In both cases the high microdiversity was correlated with assembly difficulties, and only a few long scaffolds were found for these taxa. Physiological information for these two phototrophs was based on reference genomes and isolates (Bhaya et al., 2007; van der Meer et al., 2010; Nowack et al., 2015; Olsen et al., 2015).

#### Rhodopsins

**Figure 2:**
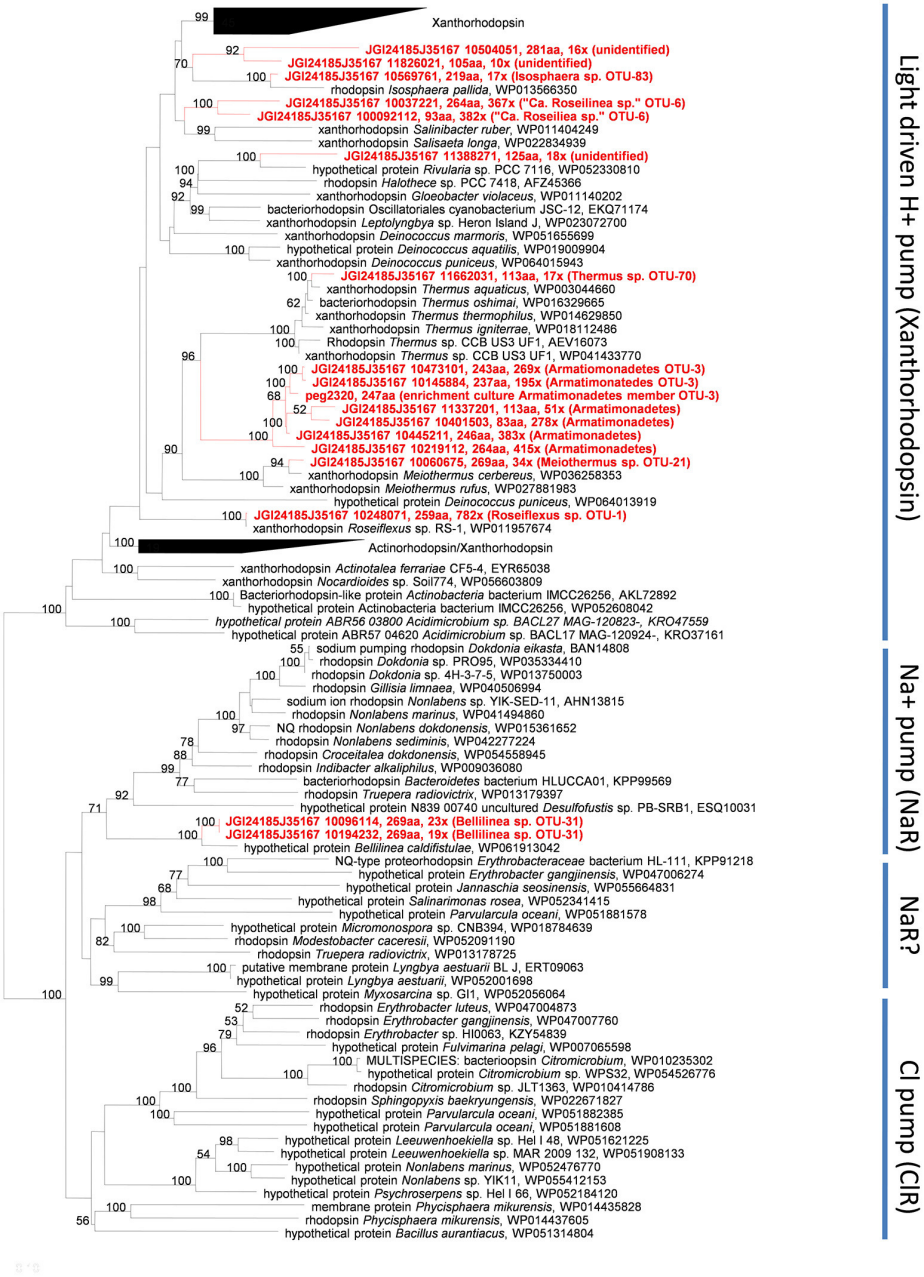
**Maximum Likelihood tree based on rhodopsin gene amino acid sequences derived from the Mushroom Spring undermat metagenome (in red) and related sequences**.

A surprising finding is that several members of the undermat contain genes belonging to the bacteriorhodopsin superfamily (Table 2). Whether these genes are used to obtain energy from light is not clear, but this is certainly a possibility. In general, blue-green light does not penetrate deeply into the mat (see Figures 4 in Nübel et al., 2002; Becraft et al., 2015), so light wavelengths absorbed by the rhodopsin-like proteins should not be abundant in the undermat. On the other hand, light of around 450–560 nm, which includes the major absorption maxima of xanthorhodopsin (Lanyi and Balashov, 2008), is not well absorbed by Chl *a*, phycocyanin and carotenoids of cyanobacteria, and thus blue-green light is slightly more abundant in deeper layers than blue or red light (Nübel et al., 2002; Becraft et al., 2015). Seventeen annotated rhodopsin genes in the assembled undermat metagenome were affiliated with seven phylogenetic groups, representing an unexpected diversity of potential retinal-based phototrophy in the mat (Figure 2; Table S3). Both the diversity and the abundance of rhodopsins was considerably lower in the upper layer. Only 10 rhodopsin sequences, representing four community members, *Roseiflexus* sp. OTU-1, *Armatimonadetes* member OTU-3 (and possibly other *Armatimonadetes* members), “*Ca*. Roseilinea gracile” OTU-6 and *Meiothermus* sp. OTU-21, were detected in the upper layer metagenome (unpublished data). Identical rhodopsin gene sequences were also detected in the undermat metagenome. It is noteworthy that all of the members showed equal (*Meiothermus* sp.) or higher relative abundance in the undermat based on 16S rRNA gene amplicon analysis, indicating retinal-based phototrophy to be of unexpected importance in a layer with low irradiance (Table 2; Thiel et al., 2016). Additionally, rhodopsin gene sequences affiliated with *Bellilinea* sp. OTU-31, *Thermus* sp. OTU-70, and *Isosphaera*-like *Planctomycetes* member OTU-83 were detected exclusively in the undermat metagenome. Most rhodopsin sequences were most closely related to genes annotated as “xanthorhodopsin,” a proton-pumping rhodopsin first described in *Salinibacter ruber*, which contains a second carotenoid chromophore (salinaxanthin) as a light-harvesting antenna molecule (Balashov et al., 2005). The presence of an antenna chromophore in xanthorhodopsin would certainly be advantageous in niches that receive low irradiance. Additional genes were identified as Na^+^-pumping rhodopsin (Figure 2).

Xanthorhodopsin genes with phylogenetic affiliation to light-driven, xanthorhodopsin-like H^+^ pumps were found in the anoxygenic, chlorophototrophic *Chloroflexi* members *Roseiflexus* spp. OTU-1 and “*Ca*. R. gracile” OTU-6. This could indicate the co-existence of xanthorhodopsin and photosynthetic apparatus, as previously suggested for the thylakoid-less cyanobacterium *Gloeobacter violaceus*, which contains gloeorhodopsin, a rhodopsin closely related to xanthorhodopsin from *Salinibacter ruber* and *Roseiflexus* sp. RS-1 (Choi et al., 2014). Rhodopsin genes in cyanobacteria do not seem to be a rare occurrence, as a keyword search in the NCBI database reveals more than 40 entries at the time of writing (March 2017). However, the absence of a carotenoid oxygenase assigned to these organisms in all datasets, the *Roseiflexus* sp. RS-1 genome, “*Ca*. Roseilinea gracile” OTU-6 partial genome and the metagenome, suggests that either a currently unidentified protein is responsible for retinal production or retinal is derived from an external source (as discussed below).

Six of the xanthorhodopsin genes in the undermat metagenome are associated with members of the phylum *Armatimonadetes* (Figure 2). Due to their high sequence similarity and similar coverage values, these sequences are probably derived from different sub-populations of the same OTU, OTU-3, for which a high microdiversity has been observed (Thiel et al., 2016). Alternatively, they could be derived from different members of the *Armatimonadetes* that are present in the mat community (e.g., OTU-3, OTU-12, and OTU-18). Whether the low-abundance xanthorhodopsin genes that cluster with sequences for *Meiothermus cerbereus* and *Thermus aquaticus* in the phylogenetic tree, really represent H^+^-pumping xanthorhodopsins is uncertain, because the rhodopsin genes in these organisms have not been studied to our knowledge nor has their functionality been verified.

The possibility of light energy usage by members of the microbial mats was further supported by a rhodopsin gene, which is phylogenetically affiliated with a less abundant member of the *Chloroflexi, Bellilinea* sp. OTU-31. Phylogenetic affiliation placed this gene within a clade of light-driven sodium pumps (Figure 2) and a similar sequence is also present in the genome of the closest relative, *Bellilinea caldifistulae*, which is a chemotrophic member of the phylum *Chloroflexi*. Although no specific function has been demonstrated for the rhodopsin gene in the type strain, when the gene obtained from the metagenome was heterologously expressed in *Escherichia coli*, the resulting strain produced a purple-colored protein and showed sodium-pumping activity (Y. Nakajima, S. Yoshizawa, V. Thiel, D.A. Bryant, unpublished results). The presence of six putative, sodium-dependent transporters in the partial genome of the *Bellilinea* sp. member in the undermat further strengthens the hypothesis that this rhodopsin has sodium-pumping activity. The evidence suggests that light-driven nutrient uptake/transport using a secondary sodium gradient, similar to the light-driven uptake of vitamin B_1_ in a marine flavobacterium (Gómez-Consarnau et al., 2016), might be important in this member of the *Chloroflexi*.

Although it is likely that the xanthorhodopsin genes described above are functional, some uncertainty exists because the partial genomes of organisms with these xanthorhodopsin genes do not generally encode a β-carotene 15,15′-monooxygenase. Xanthorhodopsin in *S. ruber* was shown to contain retinal and carotenoid salinixanthin (Balashov et al., 2005). Retinal is an oxidative cleavage product of carotenoids, most often β-carotene, which is catalyzed by a β-carotene 15,15′-monooxygenase, sometimes called β-carotene 15,15′-dioxygenase, in eukaryotes (Woggon, 2002; Kim et al., 2009). In bacteria proteins cleaving β-carotene to produce retinal include bacteriopsin-related protein (Brp) and bacteriorhodopsin-related protein-like homolog protein (Blh; (Kim et al., 2009) and references therein). Although they have the same function, mammalian β-carotene 15,15′-dioxygenase (BCD) and prokaryotic Brp/Blh family β-carotene 15,15′-monooxygenases are quite different and share only 20% amino acid sequence similarity (Sabehi et al., 2005; Kim et al., 2009). Only low-abundance sequences in the metagenome, none of which was affiliated with members containing highly abundant rhodopsin genes, showed similarity to β-carotene 15,15′-monooxygenase from *Oscillatoria nigroviridis* PCC 7112 (Table S4). Only one combination of a gene encoding an enzyme for possible cleavage of β-carotene and a xanthorhodopsin-like gene was identified for an *Isosphaera pallida*-like organism (OTU-83), a less abundant member of the undermat. This indicates the possibility of a functional xanthorhodopsin and thus light utilization for this organism. However, the failure to detect highly abundant β-carotene 15,15′-monooxygenases affiliated with more abundant rhodopsin-containing complete or partial genomes does not necessarily invalidate the hypothesis that these xanthorhodopsins are used for light energy capture. Firstly, sequences encoding enzymes similar to β-carotene 15,15′-monooxygenases, which could not be identified taxonomically, were present in the unassembled part of the metagenome. Secondly, other oxygenases could substitute for this function in different bacteria, or carotenoids other than retinal could possibly be bound to the rhodopsins. Thirdly, a still unknown family of β-carotene cleaving enzymes/genes with low sequence similarity to known carotenoid oxygenases but exhibiting the same function in other xanthorhodopsin-containing microbes might still be discovered. Finally, β-carotene 15,15′-monooxygenases that produce retinal in some of the major mat organisms (e.g., *Synechococcus* spp., *Cab. thermophilum*) might be used as a community resource by other mat members. This last possibility would be of particular ecological interest, as it would emphasize the close relationships and interactions that are connecting the mat members and stabilizing the community. Indications for this kind of co-factor dependency from the environment were recently suggested for the freshwater actinobacterium *Rhodoluna lacicola*, which expresses a functional rhodopsin gene but exhibits proton pumping activity only when exogenous retinal is provided (Keffer et al., 2015).

### Autotrophic mat members

In order to assess the autotrophic growth potential of the mat members, we analyzed key genes of different autotrophic carbon fixation pathways. Currently six autotrophic pathways are known (see Fuchs, 2011; Hügler and Sievert, 2011 for recent reviews). Four pathways, namely the Calvin-Benson-Bassham (CBB) cycle, the reverse tricarboxylic acid (rTCA) cycle, the Wood-Ljundahl (WL) pathway, and the 3-hydroxypropionate (3-HP) bi-cycle are known to occur in *Bacteria*, while the other two pathways are restricted to *Archaea* (Fuchs, 2011; Hügler and Sievert, 2011; Tang et al., 2011). Suggesting the occurrence of a quite diverse autotrophic community, genes encoding the key enzymes of all four bacterial carbon fixation pathways were detected in the undermat metagenome. This could lead to differences in carbon isotope fractionation (Hayes, 2001), although the CBB and 3-HP pathways are likely to have the most significant impact, due to the greater abundance of organisms containing these pathways.

#### Calvin-Benson-Bassham cycle

The key enzyme for the CBB cycle is the CO_2_-fixing enzyme ribulose-1,5-bisphosphate carboxylase/oxygenase (RubisCO). Almost all sequences encoding type-I RubisCO that were identified in the metagenome were affiliated with the dominant cyanobacteria, *Synechococcus* spp. Types A and B'; consistent with their known ability to fix inorganic carbon via the CBB cycle. Only one low-abundance partial sequence appeared to be affiliated with the proteobacterial phylum (JGI24185J35167_11464881; Table S5). Two low-abundance anoxygenic phototrophic *Alphaproteobacteria* were found in the undermat metagenome, “*Ca*. Elioraea thermophila” and “*Ca*. Roseovibrio tepidum” (see above). Because the genome of the closely related *Elioraea tepidum* is available and does not contain any autotrophic pathway genes, this could be an indication that a CBB cycle occurs in “*Ca*. Roseovibrio tepidum” represented by OTU-121, which is further supported by the presence of a gene for a type-1 RubisCO in the non-phototrophic but closely related *Rhodospirillales/Acetobacteraceae* member *Roseomonas gilardii subsp. rosea* ATCC BAA-691 (Acc. No. NZ_JADY01000025; L882_RS0115610).

#### 3-hydroxypropionate (3-HP) bi-cycle

The 3-HP bi-cycle is a complex autotrophic carbon fixation pathway that has been fully elucidated in *Chloroflexus aurantiacus* and most likely also operates in other autotrophic members of the *Chloroflexaceae* (Klatt et al., 2007, 2013b; Zarzycki et al., 2009; van der Meer et al., 2010). The multifunctional enzymes malonyl-CoA reductase, propionyl-CoA synthase, and malyl-CoA/β-methylmalyl-CoA/citramalyl-CoA (MMC) lyase are considered to be the key and diagnostic enzymes of the 3-HP bi-cycle (Fuchs, 2011; Hügler and Sievert, 2011 and references therein). The incorporation of CO_2_ into biomass is catalyzed by the CO_2_-fixing enzymes acetyl-CoA and propionyl-CoA carboxylase. Other characteristic enzymes are CoA-transferases and hydratases. The complete pathway is shown in Figure S1. Apart from serving as a carbon fixation pathway for autotrophic growth, the 3-HP bi-cycle may be utilized together with the TCA cycle as mixotrophic pathways for the simultaneous incorporation of organic and inorganic carbon (Klatt et al., 2007, 2013b; Zarzycki and Fuchs, 2011; Bryant et al., 2012). In nature, mixotrophy is most likely the predominant lifestyle for these organisms (also see below).

Sequences for all three of the diagnostic enzymes were detected in the metagenome for *Chloroflexi* members, the highly abundant *Roseiflexus* sp. (OTU-1), *Chloroflexus* sp. MS-G (OTU-11), and “*Ca*. Chloranaerofilum corporosum”(OTU-15), which indicates that these undermat organisms probably have the capacity to fix inorganic carbon via the 3-HP bi-cycle and grow either mixotrophically or autotrophically (Table S6).

The high microdiversity for malonyl-CoA reductase gene sequences associated with *Roseiflexus* spp. OTU-1 (Table S7) indicates the presence of putative ecotypes potentially with differences in carbon metabolism—more specifically in enzymes involved in carbon fixation. Several genes encoding important enzymes of the 3-HP bi-cycle, including acetyl-CoA carboxylase, malonyl-CoA reductase and propionyl-CoA synthase, are arranged in an apparent operon in *Roseiflexus* sp. RS-1 (RoseRS_3199–RoseRS_3203; see Klatt et al., 2007; van der Meer et al., 2010). Transcript levels for the genes in this operon increased during the day in the *Roseiflexus* spp. population in the upper green layer (Klatt et al., 2013b). A similar increase during day-time hours was observed when RoseRS_3199–3203 were used as mapping targets for RNA-seq data for the undermat (V. Thiel and D. A. Bryant, unpublished results). These data indicate that the 3-HP bi-cycle is probably active during the day when light is available and the mat is highly oxic in the upper layer.

For other members of the *Chloroflexi*, OTU-6 (“*Ca*. Roseilinea gracile”) and OTU-9 we could not detect any genes for key enzymes of the 3-HP bi-cycle (Table S6). OTU-9 instead has the genes for the reductive acetyl-CoA pathway (see below), while OTU-6 seems to have a photoheterotrophic lifestyle.

#### Reductive TCA cycle

Key enzymes of the reductive or reverse TCA cycle include 2-oxoglutarate synthase (2-oxoglutarate:ferredoxin oxidoreductase), fumarate reductase and especially the citrate-cleaving enzyme. The ATP-dependent cleavage of citrate into acetyl-CoA and oxaloacetate is a complex reaction, that can be accomplished either by the combined action of the enzymes citryl-CoA synthetase (CCS) and citryl-CoA lyase (CCL) or by the heterodimeric enzyme ATP-citrate lyase (ACL; Aoshima, 2007; Hügler et al., 2007; Hügler and Sievert, 2011). Sequences coding for ACL [either the conventional type I, or the novel proposed type II (Hügler and Sievert, 2011; Thiel et al., 2012)] were not found in the metagenome. However, genes encoding the two-enzyme variant of citrate cleavage were present in the metagenome and could be assigned to two mat members. Two copies each of the genes encoding CCS and CCL were affiliated with a highly abundant mat member, *Thermocrinis* sp. OTU-4, which supports the presence of at least two different populations for this taxon in the mat as previously discussed (Thiel et al., 2016). Chemolithoautotrophic growth has been shown to occur for the closest relative, *Thermocrinis ruber* with H_2_, thiosulfate and S^0^ as electron donors and oxygen as electron acceptor (Huber et al., 1998; Hügler et al., 2007). In addition to CCS and CCL, all other genes necessary for a functional reductive citric acid cycle in *Thermocrinis* sp. OTU-4 could be identified in the metagenome (see Figure S2). In addition to the two-step citrate cleavage mechanism, *Thermocrinis* sp. OTU-4 also accomplishes a two-step carboxylation of 2-oxoglutarate to isocitrate using the enzymes 2-oxoglutarate carboxylase and oxalosuccinate reductase instead of the conventional isocitrate dehydrogenase. Both of these two-step reactions, the two-step citrate cleavage, as well as the two-step carboxylation of 2-oxoglutarate, might be relics of an ancient variant of the reductive TCA cycle, that has been shown to occur in other members of the family *Aquificaceae*, e.g., *Hydrogenobacter thermophilus* or *Aquifex aeolicus* (Aoshima, 2007; Hügler et al., 2007).

**Figure 3:**
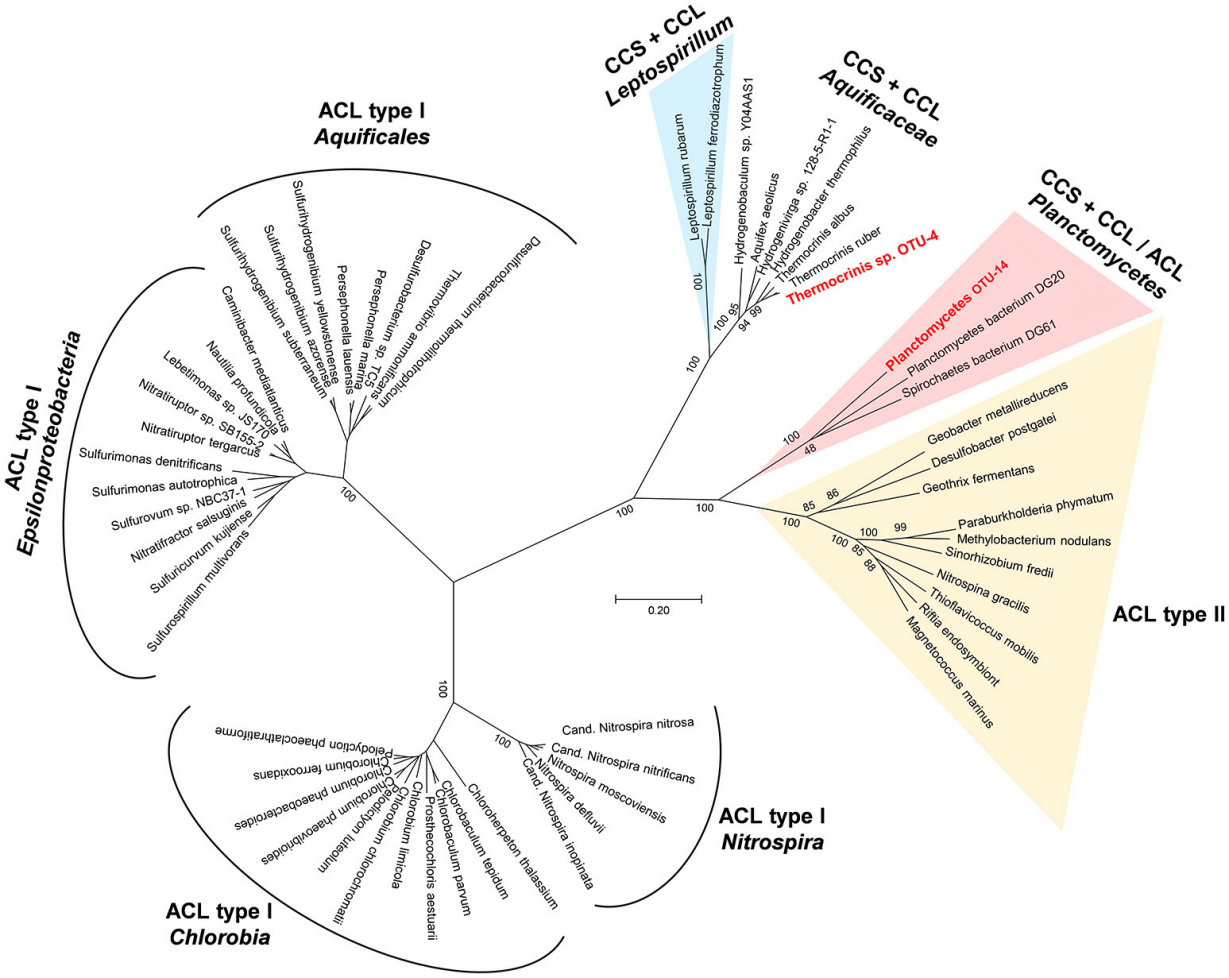
**Phylogenetic tree showing the relationship of protein sequences identified in the undermat metagenome (in red) as Types I and II ACL (ATP dependent citrate lyase) as well as citryl-CoA synthetase (CCS) and citryl-CoA lyase (CCL) protein sequences from the public databases**.

Another set of genes potentially encoding the citrate-cleaving enzymes CCS and CCL were found on scaffold JGI24185J35167_1000715, which is part of a metagenomic bin identified as OTU-14, a member of the *Planctomycetes*. The genes were annotated as “succinyl-CoA synthetase alpha and beta subunit” and “citrate synthase” (JGI24185J35167_10007157-9). BLASTp searches against the nr database identified hypothetical proteins from *Spirochaetes, Phycisphaerae* and *Plantomycetes* as closest relatives with amino acid sequence identity values of 52–54%. A CDD search identified the genes as “CS_ACL-C_CCL superfamily” citrate synthase as well as “succinyl CoA synthetase alpha and beta subunits.” A phylogenetic analysis using the amino acid sequences of both subunits of ACL and the amino acid sequencing of the concatenated enzymes CCS and CCL is shown in Figure 3. The putative citrate cleaving genes of OTU-14 and related genes from other *Plantomycetes* cluster between the CCS-CCL of the *Aquificae* and *Leptospirillum* and ACL type II sequences. Thus, the genes for citrate-cleaving enzymes of these *Planctomycetes* bacteria give important insights into the evolution of the reductive TCA cycle and ACL. Phylogenetically, these enzymes are intermediates between the ancient variant that is still present within the *Aquificaceae* and the type II ACL (Figure 3). Interestingly, only OTU-14 shows separate genes for CCS and CCL, while the closest sequences from the database (e.g., *Planctomycetes* bacterium DG20) has a heterodimeric ACL; the gene for the second subunit of CCS (*ccsB*) and *ccl* are fused to form one gene, *aclA* (Figure S3). A possible explanation for this difference would be temperature. While OTU-14 is a thermophile (water temperature above the mat was 60°C; Thiel et al., 2016), the closely related *Planctomycetes* DG20 was detected in a metagenomics study of estuary sediments from North Carolina (Baker et al., 2015), and thus lives in a mesophilic environment. Another surprising feature of the bin assigned to *Planctomycetes* sp. OTU-14 is, that in addition to the key genes of the reductive TCA cycle, it also harbors genes encoding carbon monoxide dehydrogenase/acetyl CoA synthase and other enzymes of the reductive acetyl-CoA pathway. Thus, this organism has the genomic potential to use two different carbon fixation pathways for autotrophic growth (see below).

The reductive carboxylation of either succinyl-CoA or acetyl-CoA to 2-oxoglutarate or pyruvate, respectively, is carried out by the enzymes 2-oxoglutarate or pyruvate:ferredoxin oxidoreductases. Although necessary for autotrophic bacteria using the reductive TCA cycle for carbon fixation, these enzymes can also occur in heterotrophic or mixotrophic microbes. These enzymes perform anaplerotic CO_2_ fixation reactions that may play an important role within the undermat portion of the community, as genes encoding several versions of these enzymes are found in the metagenome. Thus, several undermat organisms possibly require an ample supply of ${\text{HCO}}_{3}^{-}$/CO_2_ for biosynthetic purposes as has been shown for *Cab. thermophilum* (Tank and Bryant, 2015a,b) and hypothesized for “*Ca*. T. aerophilum” (Liu et al., 2012).

#### Wood-Ljungdahl pathway

The reductive acetyl-CoA or Wood-Ljungdahl (WL) pathway is the only known carbon fixation pathway used by *Bacteria* as well as by *Archaea*, consistent with the hypothesis that it is the most ancient pathway (e.g., Weiss et al., 2016). The key enzyme of the pathway is CO dehydrogenase/acetyl-CoA synthase (CODH/ACS), which catalyzes the reduction of CO_2_ to CO as well as the synthesis of acetyl-CoA from the methyl- and carbonyl moieties. The reduction of CO_2_ to the methyl group is accomplished by enzymes that are also characteristic for this pathway (Figure S4; see Ragsdale and Pierce, 2008 for a review).

Four potentially bi-functional CODH/ACS subunit genes were identified in the metagenome (Table S8). The most abundant putative CODH/ACS gene (124 × coverage) belonged to the metagenomic bin for OTU-9, which has been associated with an *Anaerolineae*-like chemotrophic member of the *Chloroflexi* (Thiel et al., 2016). The protein shows highest similarity (55%) to CODH/ACS genes from *Deltaproteobacteria* and *Clostridia* (e.g., *Desulfonatronospira* sp., *Ammonifex degensii*) for which autotrophic growth has been observed, and enzyme activity measurements strongly suggest an active reductive acetyl-CoA pathway (Sorokin et al., 2008). Thus, the metagenomic data suggests that the *Anaerolineae*-like member of the *Chloroflexi* OTU-9 has the potential to grow autotrophically via the WL pathway. The next most abundant CODH/ACS sequences had read depths of 68× and 24×; these sequences belonged to two different potential members of the *Planctomycetes*, one representing OTU-14 (see below), and the other a less abundant member of the undermat layer that was not identified by 16S rRNA (Thiel et al., 2016). The least abundant CODH/ACS gene belonged to a member of the *Thermodesulfobacteriaceae* (OTU-26), a relative of *Caldimicrobium thiodismutans*, for which autotrophic growth by hydrogen oxidation or disproportionation of sulfur compounds has been demonstrated (Kojima et al., 2016). Two copies of CODH/ACS sequences were affiliated with *Thermodesulfovibrio* sp. OTU-8, a member of the phylum *Nitrospirae*. In addition to that, the genes for all other enzymes of the reductive acetyl-CoA pathway were found in the metagenomic bin representing OTU-8. Similarly, the genes for the reductive acetyl-CoA pathway were also present in the genome of the closely related species *Thermodesulfovibrio yellowstonii* (Table S8). Many acetotrophic, sulfate-reducing bacteria employ the WL pathway to cleave acetyl-CoA. Yet, the presence of a bifunctional CODH/ACS suggests, that *Thermodesulfovibrio* spp. are at least facultative autotrophs, although autotrophic growth has not been demonstrated for *T. yellowstonii* (Henry et al., 1994).

The most intriguing finding is the presence of genes encoding a bifunctional CODH/ACS together with genes for other enzymes of the reductive acetyl-CoA pathway in the *Planctomycetes* sp. OTU-14, in addition to genes of the reductive TCA cycle. We checked the metagenome of the closely related *Planctomycetes* sp. DG20 and also found the genes for the bifunctional CODH/ACS. Thus, these uncultured members of the *Planctomycetes* encode the genes for enzymes of two carbon fixation pathways, the reductive TCA cycle and the reductive acetyl-CoA pathway. So far, the presence of two different carbon fixation pathways in one bacterium has been only been described for the endosymbionts of vestimentiferan tubeworms living near hydrothermal vents and seeps (Markert et al., 2007; Thiel et al., 2012). This endosymbiotic gammaproteobacterium uses the reductive TCA cycle in addition to the CBB cycle for carbon fixation. As mentioned above, the reductive acetyl-CoA is most likely the most ancient autotrophic carbon fixation pathway (Sojo et al., 2016; Weiss et al., 2016). Braakman and Smith (2012) proposed that a hybrid reductive TCA/ reductive acetyl-CoA pathway is the basis of all extant carbon fixation pathways. A more detailed study of the genomes of these members of the *Planctomycetes* might shed more light on the early evolution of carbon fixation.

### Heterotrophic lifestyle

**Figure 4:**
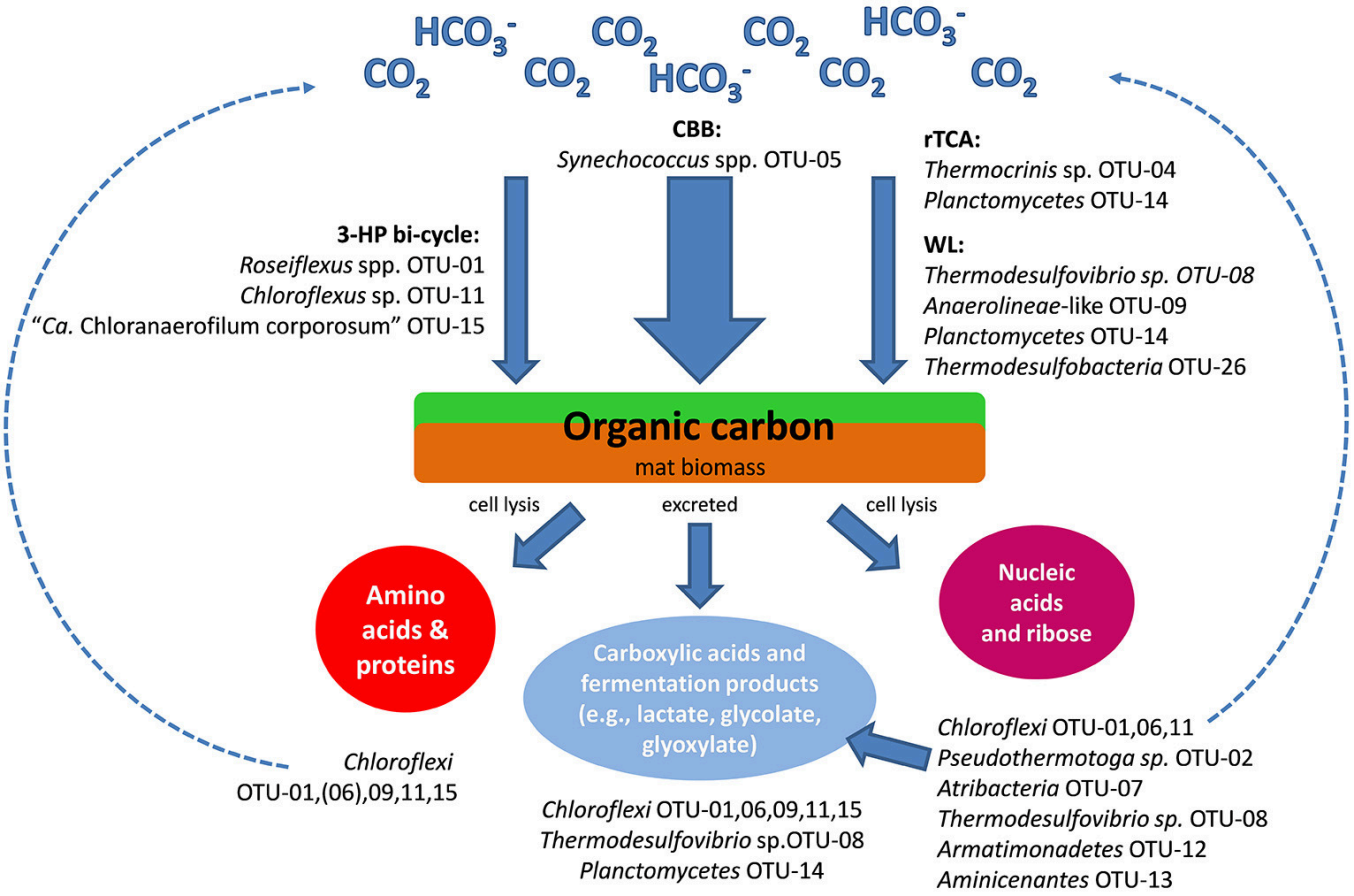
**Schematic drawing of the hypothesized carbon cycle and the likely involved mat community members as inferred from analyses of metagenomic gene clusters**. CBB, Calvin-Benson-Bassham cycle; rTCA, reverse tricarboxylic acid cycle; WL, Wood-Ljungdahl pathway; 3-HP bi-cycle - 3-hydroxypropionate bi-cycle.

Five of the 15 most abundant microbial undermat members were identified as chlorophototrophic bacteria, but only one group, the dominant cyanobacteria of the upper green layer, are photoautotrophs with regard to carbon fixation. In addition, the capacity for chemolithoautotrophic growth can be assumed for *Thermocrinis* sp. OTU-4, and putatively for *Planctomycetes* member OTU-14, *Chloroflexi* member OTU-9 and *Thermodesulfovibrio* sp. OTU-8. All other members of the mat probably grow photomixotrophically (e.g., *Roseiflexus* sp. OTU-1, *Chloroflexus* sp. OTU-11, and “*Ca*. Chloranaerofilum corporosum” OTU-15) or heterotropically, and thus they benefit from or depend on organic carbon sources provided by the primary producers (Table 3) by excretion or cell lysis (Figure 4).

**Table 3:** **List of the 15 most abundant undermat members (based on 16S rRNA gene amplicon sequence abundance) their relative abundance, predicted metabolic life style, and relationship to oxygen**.

**OTU**	**Identity**	Rel. abundance^*^ (%)	**Predicted metabolic lifestyle**	**Relationship to oxygen**
OTU-01	*Chloroflexi, Roseiflexus* sp.	49	Anoxygenic photomixotroph	Oxygen tolerant
OTU-02	*Thermotogae, Pseudothermotoga* sp.	10	Chemoheterotroph, fermentation	Anaerobe
OTU-03	*Armatimonadetes* (OP10) (OS Type L)	4	Chemoheterotroph, respiration, fermentation	Facultative anaerobe
OTU-04	*Aquificae, Thermocrinis* sp.	4	Chemoautotroph, sulfur-oxidation	Aerobe
OTU-05	*Cyanobacteria, Synechococcus* sp. B'	4	Oxygenic photoautotroph	Aerobe
OTU-06	*Chloroflexi, Ca*. Roseilinea gracile	3	Anoxygenic photoheterotroph	Aerobe
OTU-07	*Atribacteria* (OP9)	2	Chemoheterotroph, fermentation	Anaerobe
OTU-08	*Nitrospirae, Thermodesulfovibrio* sp.	2	Dissimilatory sulfate-reduction	Anaerobe
OTU-09	*Chloroflexi, Anaerolinea*-like	1	Chemoautotroph	Anaerobe
OTU-10	*Themotogae*, EM3	1	Chemoheterotroph, respiration	Aerobe
OTU-11	*Chloroflexi, Chloroflexus* sp.	1	Anoxygenic photoauto/mixotroph	Oxygen-tolerant
OTU-12	*Armatimonadetes* (OP10), Group 6	1	Chemoheterotroph, anaerobe respiration	Anaerobe
OTU-13	*Aminicenantes* (OP8)	1	Chemoheterotroph, fermentation	Anaerobe
OTU-14	*Planctomycetes*	1	Chemoautotroph	Facultative anaerobe
OTU-15	*Chloroflexi*, “*Ca*. Chloranaerofilum corporosum”	1	Anoxygenic photoauto/mixotroph	Strict anaerobe

* *Based on 16S rRNA amplicon sequence abundance (Thiel et al., 2016)*.

Both aerobic and anaerobic respiration, as well as fermentative, heterotrophic lifestyles are indicated for the different members of the mat community (Table 3). In addition to cellular biopolymers, e.g., nucleic acids and proteins, carboxylic acids, and fermentation products are available to the heterotrophic mat community (Kim et al., 2015). Photoassimilation of fermentation products, such as acetate, glycolate, propionate and lactate, by phototrophic *Chloroflexi* has been demonstrated both in pure cultures as well as under *in situ* conditions, and the exchange of glycolate and lactate between *Synechococcus* spp. and *Roseiflexus* spp. in the microbial mats has been further supported by metabolomic and metatranscriptomic studies (Anderson et al., 1987; Bateson and Ward, 1988; Liu et al., 2012; Kim et al., 2015). Bulk protein and amino acids have been indicated to play an important role in nutrient cycling in the mat (see below). Amino acids and protein are used by *Cab. thermophilum*, and presumably also by “*Ca*. Thermochlorobacter aerophila,” not only as N-sources but also as the principal C-source (Liu et al., 2012; Tank and Bryant, 2015a,b; Tank M, unpublished data). Beyond this, little is presently known about the pathways of nutrient and carbon cycling within the mats. The metagenome and the growing number of isolates should allow one to examine the genetic potential of the heterotrophic mat members and determine more details about carbon and nutrient cycling in the mats.

#### Lactate utilization

Lactate is present in the microbial mat in the late afternoon, and it has been suggested that it is exchanged between *Synechococcus* and *Roseiflexus* spp. (Kim et al., 2015). Lactate permease genes are present in the genome(s) of *Roseiflexus* spp.; their relative transcript levels become more abundant during the early evening hours, and lactate levels decline after this occurs. The ability to take up and utilize lactate, as indicated by the presence of genes encoding lactate permease and lactate dehydrogenase genes, is indicated for at least four and up to 7 of the 15 most abundant undermat members (Table S9). Additionally, genes encoding lactate permease are affiliated with some less abundant members, e.g., *Thermodesulfobacteria* sp. OTU-26, *Bellilinea* sp. OTU-31, *Solibacter-*like *Acidobacteria* OTU-36, and “*Ca*. E. thermophila” OTU-46.

Due to the high abundance and activity of cyanobacterial cells in the upper green layer of the mat, the measured and available lactate has mostly been attributed to *Synechococcus* spp. Types A and B' (Kim et al., 2015). Metagenomic data from the undermat layer, however, indicates additional putative producers of lactate as a fermentation end-product: e.g., *Armatimonadetes* (OP10) member OTU-3, fermentative *Atribacteria* (OP9) member OTU-7 and *Aminicenantes* (OP8) member OTU-13 (Table S9).

#### Saprophytic life style

A saprophytic life style, based on cellular components made available by cell lysis of the major primary producer, *Synechococcus* spp., can be assumed for the majority of the heterotrophic mat community. Excreted proteases, secretion systems, and pathogenesis islands were not observed, so there was little evidence for an active predatory life style for any of the most abundant undermat members, neither in the metagenome nor in the reference genomes employed in this study (data not shown). The presence of viruses in the mat and their probable involvement in cell lysis processes can be assumed (e.g., on the basis of CRISPR systems in genomes), but this has not yet been analyzed and must be shown by future studies.

#### Protein and amino acids

The presence of genes for transporters for oligopeptides and dipeptides, as well as generic amino acid and/or branched chain amino acid (BCAAs) transporters, were identified in the majority of the abundant mat members. This suggests that most organisms in the undermat have the potential to utilize peptides and amino acids as sources of carbon and nitrogen. Nearly complete degradation pathways for BCAAs were found for all four abundant *Choroflexi* members (Table S10). This indicates the ability to utilize BCAAs as putative carbon sources for these organisms, which is further supported by the ability of the chlorophototrophs to grow in CTM-medium that contains BCAAs (Tank and Bryant, 2015a,b; Tank et al., 2017). However, complete dependency on BCAAs, as demonstrated for *Cab. thermophilum* (Tank and Bryant, 2015a,b) and postulated for “*Ca*. Thermochlorobacter aerophilum,” was generally not found except for perhaps *Chloroflexi* member OTU-9, for which the partial genome so far only indicates biosynthesis of BCAAs from the precursor 2-isopropylmalate. Degradation of BCAAs is also likely to occur for *Armatimonadetes* member OTU-3 and *EM3* member OTU-10 (Table S10).

#### Nucleic acids

Utilization of nucleic acids as a nutrient and energy source is suggested to occur in these mats. For example, at least 13 of the 15 most abundant members should be able to utilize ribose, as indicated by the presence of genes for both ribokinase and ribose-5-phosphate isomerase in metagenomic bins and/or reference genomes (Table S11). The presence of ABC-type ribose transporter genes in at least six of undermat members further supports the uptake and utilization of ribose from the environment by these organisms. Furthermore, 23 predicted extracellular nucleases were present in the metagenome, of which 10 were affiliated with 4 of the most abundant members (Table S11). Two highly abundant genes could not be affiliated with a specific taxon (JGI24185J35167_10498681, JGI24185J35167_10496441). Thus, up to eight highly abundant mat members may be able to degrade extracellular nucleic acids and utilization the resulting nucleotides, phosphate, and/or ribose.

### Nitrogen metabolism

**Figure 5:**
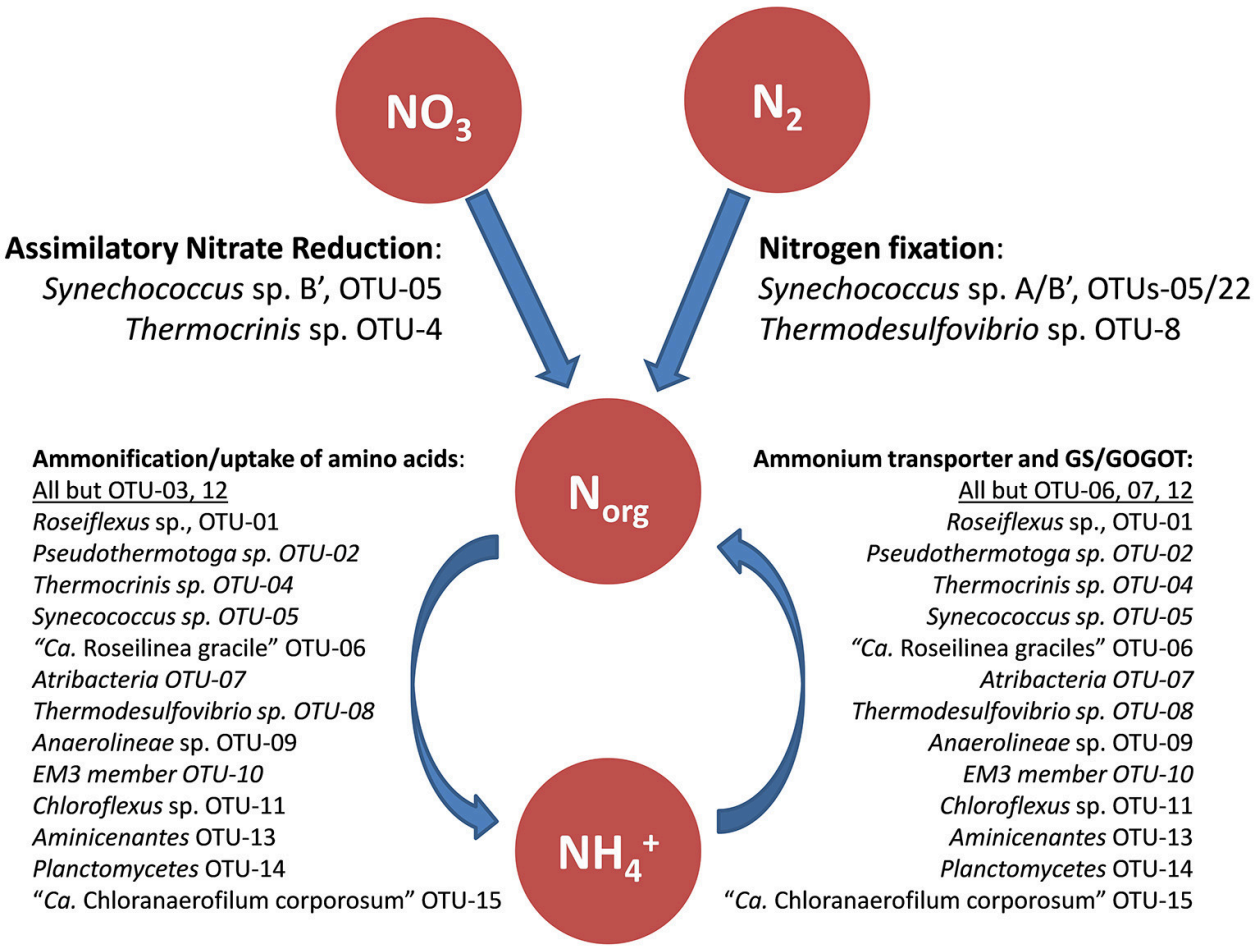
**Schematic drawing of the hypothesized nitrogen metabolism in the mat including likely involved mat community members**. GS, Glutamine synthetase; GOGAT, glutamate synthase.

Nitrogen metabolism in the mat seems to be purely assimilatory, and no genes suggesting dissimilation of nitrogen compounds were identified in the metagenome (Figure 5; Table S12). Organic nitrogen compounds produced from nitrogen fixation by *Synechococcus* spp., and probably by *Thermodesulfovibrio* sp., provide the mat community with reduced nitrogen (e.g., amino acids, nucleic acids, and ammonia). It is possible that some organisms in the mat can use the low levels of ammonium in the spring water (Thiel et al., 2016) as a source of reduced nitrogen. In this respect, nitrate and ammonium do not stimulate the growth of *Synechococcus* spp. in enrichment cultures (M. Tank, personal communication).

#### Nitrogen fixation

Genes encoding putative nitrogenases (*nifHDK*) were found in four members of the mat: *Synechococcus* spp., *Roseiflexus* spp., “*Ca*. Chloranaerofilum corporosum” and *Thermodesulfovibrio* sp. (Table S12). However, active nitrogen fixation has only been shown for one of these organisms, *Synechococcus* spp. (Steunou et al., 2006, 2008), which are believed to provide the mat community with bio-available fixed nitrogen. It is not clear what the function of the *nifHDK*-like genes in *Roseiflexus* spp. might be, because most other accessory proteins required for maturation of nitrogenase are not present in any available *Roseiflexus* spp. genome (only *nifB* is present), and e.g., *Roseiflexus castenholzii* is unable to grow on dinitrogen as sole nitrogen source (D.A. Bryant, unpublished results). “*Ca*. Chloranaerofilum corporosum” contains the same set of genes as *Roseiflexus* spp. and is similarly not expected to grow on dinitrogen as sole nitrogen source. Frank et al. (2016) have reported that three *Thermodesulfovibrio* spp., *T. yellowstonii, T. aggregans*, and *T. islandicus*, genomes have the complete set of genes for nitrogenase, strongly suggesting that these organsims use dinitrogen as a nitrogen source; however, diazotrophic growth has not been reported for any of these isolates. The presence of additional *nif* genes (*nifENXB*) as well as a transcriptional repressor of the *nif* operon, suggests that this *Thermodesulfovibrio* sp. is also likely to be a nitrogen-fixing organism. This suggestion is strengthened by the observation that transcription of the *nif* genes in *Thermodesulfovibrio* sp. occurs and varies as a function of the diel cycle (unpublished observations). The presence of similar organisms in other hot spring microbial mats, especially in those dominated by non-nitrogen fixing cyanobacteria (Everroad et al., 2012), supports the possibility that they have the ability to fix nitrogen *in situ* and thus contribute to nitrogen input to the microbial mat community.

#### Assimilatory use of ammonium

Despite the low concentration of ammonium in the hot spring water (values ranging from <3 to 35 μM have been measured over the years; Doemel and Brock, 1976; Papke et al., 2003; Ball et al., 2004), ammonium seems to be the preferred nitrogen source, along with organic nitrogen compounds such as amino acids (see above). Ammonium probably becomes available through ammonification and through the degradation of organic compounds, specifically of cyanobacterial proteins and nucleic acids. In accordance with this, numerous annotated genes for ammonium permease (*amtB*) are found in the undermat metagenome (95 partial genes in total). Putative ammonium permease genes were identified in 10 of the 15 most abundant mat members (Table S12). Genes for the key enzymes utilized in both ammonification (amino acid catabolism) and use of ammonium in (amino acid) anabolism, glutamine synthetase (GS), and glutamate:2-oxoglutarate amidotransferase (GOGAT; glutamate synthase) are present in high numbers in the metagenome.

#### Nitrate reduction

Based on the metagenomic data, nitrate reduction appears to be uncommon in the microbial undermat layer. Although genes encoding nitrite reductase (*nirB*) and nitrate reductase (*narB)* in combination with genes for a nitrate ABC transporter (*nrtABC*), are present in 2 of the 15 most abundant mat members (Table S12), assimilatory nitrate reduction is only assumed to occur in *Thermocrinis* sp. OTU-4.

Based on the presence of *nirA* and *narB* genes in the genomes of isolates as well as the metagenome, assimilatory nitrate reduction has long been assumed to occur for the dominant *Synechococcus* spp. Moreover, nitrate has traditionally been the only nitrogen source used in cultivation experiments of uni-cyanobacterial mixed cultures of *Synechococcus* spp. Types A and B' obtained from the mats (Nowack et al., 2015; Olsen et al., 2015). However, in experiments with *Synechococcus* spp. isolates on agar plates aiming to produce axenic cultures, nitrate did not support growth under constant light and oxygen conditions nor did it enhance growth under conditions that favor nitrogen fixation. Thus, nitrate may not be assimilated by *Synechococcus* spp. under laboratory conditions (M. Tank, N. Soulier, D.A. Bryant, unpublished). These observations might be explained by micro-niche conditions favoring nitrogen fixation or a nitrate-reducing “helper” bacterium present in these mixed cultures. In contrast, assimilatory nitrate reduction can be assumed for *Thermocrinis* sp. OTU-4, for which *nirA* and *narB* genes showing closest similarity to *Thermocrinis ruber* were identified in the metagenome. Assimilatory nitrate reduction for this organism is further supported by chemolithoautotrophic growth of its closest relative, *Thermocrinis ruber*, in media containing nitrate as sole nitrogen source (Härtig et al., 2014). Similarly, the absence of *narGHI* genes in the genome of the type strain, as well as the metagenome sequences, correlate with the observation that nitrate is not an electron acceptor that can support the growth of *Thermocrinis ruber* strain OC1/4 (Huber et al., 1998).

#### Nitrification and denitrification

Nitrification does not seem to occur in any of the abundant microbial undermat members. Ammonia monooxygenase (*amoA*), the key enzyme responsible for the ammonium oxidation step in nitrification, was not identified in the metagenome. Similarly, dentrification genes were not detected in the undermat metagenome. Therefore, denitrification does not appear to occur in any abundant mat organism. This is consistent with the low concentration of nitrate in the spring water (<0.1 mg/L; ~ <1.6 μM; see Thiel et al., 2016).

### Sulfur metabolism

**Figure 6:**
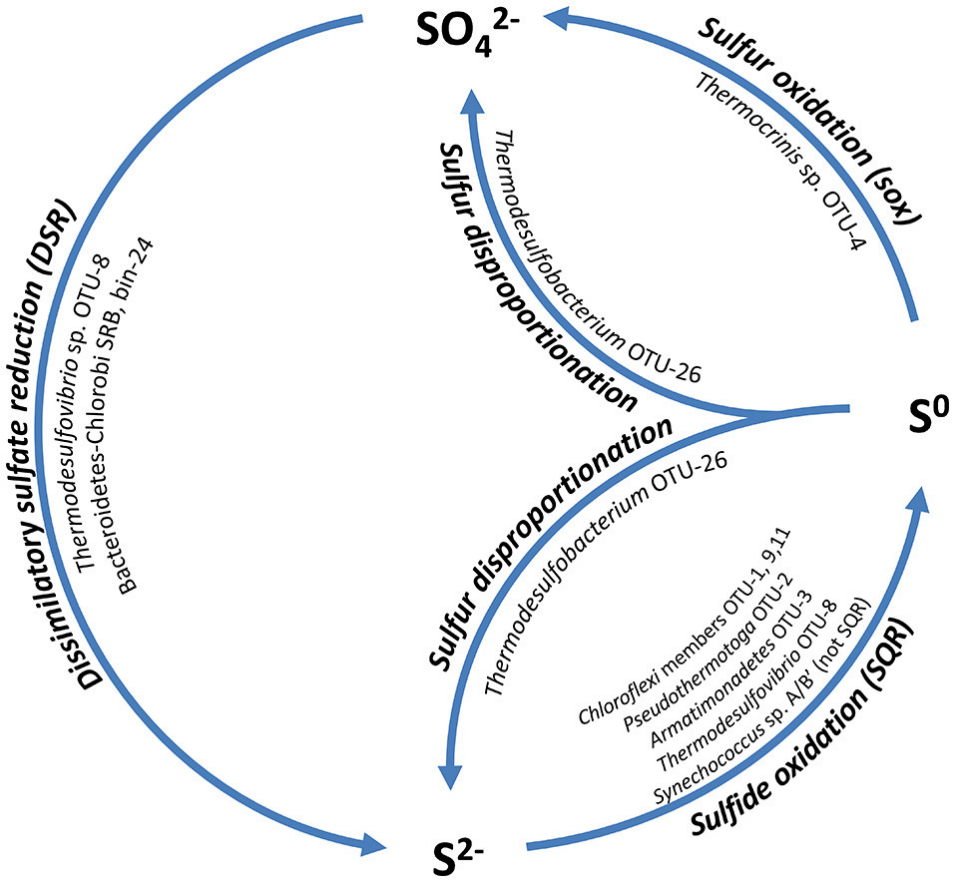
**Schematic drawing of the hypothesized sulfur cycle and the involved mat community members**. SQR, Sulfide:quinone oxidoreductase; sox, genes for sulfur oxidation pathway; DSR, dissimilatory sulfate reductase.

Despite the low sulfate concentration in the spring water (<200 μM; USGS report 2001–2002 Ball et al., 2004; Dillon et al., 2007), an active sulfur cycle is likely maintained in the mats (Figure 6). Sulfate reduction has previously been shown in the mats (Dillon et al., 2007), while the oxidation of sulfide and sulfur has not been studied in detail. Metagenomic analysis of the undermat supports the hypothesis of a closed sulfur cycle and identified the key organisms involved.

#### Sulfate reduction

Dissimilatory sulfate reduction is likely to occur in at least three members of the mat community. Previous studies revealed four distinct clusters of *dsrAB* sequences by a PCR-based cloning survey (Dillon et al., 2007). Two complete sets of sulfate reduction genes (*sat, aprBA, dsrAB, dsrTMKJOP, qmoABC*) were present in the metagenomic dataset. The more abundant set of genes (~127 × coverage for the *dsrAB* genes) belonged to *Thermodesulfovibrio* sp. OTU-8, which was associated with measured sulfate reduction activity in previous studies (“clade-1,” Dillon et al., 2007). A second set of less abundant *dsrAB* genes (63 × coverage) were the same as unidentified *drsAB* clone sequences that were previously retrieved from the mats (“clade-2,” Dillon et al., 2007). These sequences were affiliated with a metagenomic bin (bin-24, Thiel et al., 2016), with an uncertain phylogenetic affiliation within the *Bacteroidetes-Chlorobi* group. Due to the lack of a 16S rRNA gene sequence in the metagenome bin, phylogenetic affiliation was based on phylogenetic marker genes, most of which were affiliated with either the *Bacteroidetes* or the *Chlorobi* group. Phylogenetic analyses of 17 concatenated phylogenetic marker genes placed the unidentified novel uncultured organism in a cluster with *Bacteroidetes* and both heterotrophic as well as phototrophic *Chlorobi* representatives (Thiel et al., in preparation). Furthermore, the presence of a 1023-bp partial 23S rRNA gene sequence with closest relationship to *Bacteroidetes-Chlorobi* group members strengthens the affiliation. “Clade-3” sequences were also found in the metagenome at very low abundance (6–8×) (Table S13). A previous phylogenetic analysis by Müller et al. (2015), as well as this study, places these sequences into an unclassified cluster within a group of *Firmicutes*-like *dsrAB* sequences (*dsrAB* ARB database available at http://www.microbial-ecology.net/download). “Clade-4” sequences reported by Dillon et al. (2007) were not found in the undermat metagenome.

In addition to the previously known *dsrAB* phylotypes, sequences encoding dissimilatory sulfite reductase from a *Thermodesulfobacteria* member, putatively OTU-26, were recovered from the metagenome. Three low abundance partial *dsrAB* sequences (16–26 × coverage) showed closest similarity to *Caldimicrobium thiodismutans* with 87–93% amino acid identity and 96–98% similarity (Table S13). *C. thiodismutans* has recently been isolated from a hot spring in Japan and displays sulfur disproportionating activity (Kojima et al., 2016). A similar sulfur-disproportionating metabolism is hypothesized for the Mushroom Spring mat member (Figure 6).

Assimilatory sulfate reduction does not seem to be prevalent in the undermat metagenome. This indicates that the majority of mat members rely on reduced sulfur sources, as has been shown for *Chloracidobacterium thermophilum* (Tank and Bryant, 2015a,b). Genes for ferredoxin-dependent sulfite reductase (*sir*) were only identified for 3 of the 15 most abundant undermat members (OTUs-5, 11, and 14; Table S14). Additional low-coverage gene sequences were affiliated with less abundant members, e.g., *Armatimonadetes* member OTU-18, *Acidobacterium* member OTU-36 (bin-20, Thiel et al., 2016) as well as two unidentified *Planctomycetes* members (bins-23 and 29; Thiel et al., 2016), while some remained unidentified. *Roseiflexus* spp. strains do not contain complete reduction pathways and presumably cannot perform assimilatory sulfate reduction (Bryant et al., 2012). Among members of the phylum *Chloroflexi*, genes for sulfite reductase were only identified in *Chloroflexus* sp. OTU-11.

#### Sulfur oxidation

Although the concentration of sulfide present in the hot spring source water is at the limit of detection (0.003 mg/L = ~0.1 μM, Ball et al., 2004), sulfide accumulates in the mats as a result of biological sulfate reduction (Dillon et al., 2007). Little is known about the oxidative part of the sulfur cycle in the mats. Sulfide can be used as an electron donor by some anoxygenic phototrophic bacteria, but it is also toxic to many organisms, and detoxification mechanisms have evolved. *Chloroflexi* are known to contain sulfide:quinone oxidoreductase (*sqr*) of the type II family (Bryant et al., 2012) and have the ability to oxidize sulfide to polysulfides (Madigan and Brock, 1975), which sometimes even support autotrophic growth (Madigan et al., 1974; Madigan and Brock, 1977; Keppen et al., 2000; Gich et al., 2003; Klappenbach and Pierson, 2004; Thiel et al., 2014). SQR genes were also found in the metagenome and were affiliated with chlorophototrophic and chemotrophic members of the *Chloroflexi, Thermotogae, Armatimonadetes*, and *Nitrospirae* (Table S14).

When sulfide is oxidized by *Chloroflexus* and *Oscillochloris* spp., globules of elemental sulfur are deposited outside the cells (Madigan and Brock, 1975; Keppen et al., 2000). Elemental sulfur was shown to be a sulfur source for the growth of *Chloracidobacterium thermophilum* (Tank and Bryant, 2015a,b). It might also be a sulfur source for other mat members that lack the genes for assimilatory sulfate reduction (e.g., OTU-7, see Table S14). Furthermore, elemental sulfur is known to be an electron donor for *Thermocrinis ruber* (Huber et al., 1998), which is the closest type-strain relative to the microbial mat member *Thermocrinis* sp. OTU-4 (Thiel et al., 2016), for which a complete set of *sox* genes was identified in the metagenome. It is thus expected that this mat member can oxidize elemental sulfur to sulfate, which would close the sulfur cycle and provide sulfate that can act as electron acceptor for biological sulfate-reduction by *Thermodesulfovibrio* sp. OTU-8 and any other putative sulfate-reducers in the mat (Figure 6).

There is evidence that *Synechococcus* spp. in the mats can also oxidize sulfide. *In situ* studies showed that sulfide addition strongly stimulated ^13^C-bicarbonate incorporation into *Synechococcus* spp. lipid biomarkers (van der Meer et al., 2005). Some cyanobacteria are known to possess sulfide quinone reductase, but this gene is not present in the genomes of *Synechococcus* spp. that occur in the mats. Cyanobacteria that do not produce polysulfides from sulfide oxidation produce thiosulfate as the product (De Wit and van Gemerden, 1987; Rabenstein et al., 1995); however, the mechanism of thiosulfate production by such organisms is currently unknown.

### Hydrogen metabolism

**Figure 7:**
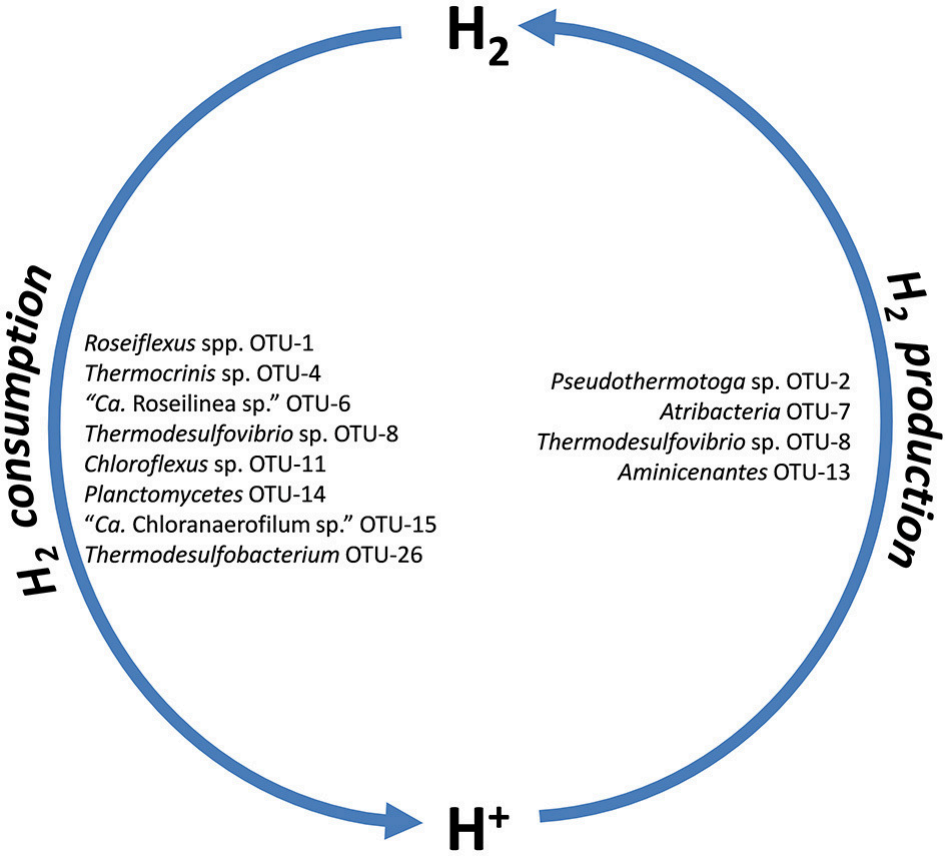
**Putative H_**2**_ producers and consumers of the microbial mat community as inferred from metagenome analysis**.

Revsbech et al. (2016) have recently studied the diel H_2_ dynamics in the Mushroom Spring mat. Light-stimulated H_2_ production as a by-product of cyanobacterial N_2_ fixation was shown to occur in the morning, while H_2_ accumulated in the evening and slowly decreased until just before sunrise. Metagenomic analyses indicate fermentative sources for the production of H_2_ in the evening and night by *Pseudothermotoga* sp., *Thermodesulfovibrio* sp. and members of the *Atribacteria* and *Aminicenantes* (Figure 7). H_2_ levels during the day are low (Kim et al., 2015; Revsbech et al., 2016), which presumably is due to its oxidation by chlorophototrophic members of the *Chloroflexi* as well as *Thermocrinis* sp., *Thermodesulfovibrio* sp., *Planctomycetes*, and *Thermodesulfobacteria* (Table S15, Figure 7).

#### H_2_ production

Microbes can produce molecular hydrogen (H_2_) via fermentation and dinitrogen fixation (Nielsen et al., 2015; Revsbech et al., 2016). Hydrogen accumulation has been detected in these mats following a diel pattern. In the evening, H_2_ accumulated rapidly after the onset of darkness, reaching peak values of up to 30 μmol H_2_ L^−1^ at about 1-mm depth below the mat surface, and slowly decreasing to about 11 μmol H_2_ L^−1^ just before sunrise (Revsbech et al., 2016). Another pulse of H_2_ production, reaching a peak concentration of 46 μmol H_2_ L^−1^, was found in the early morning under dim light conditions that were too low to cause the accumulation of O_2_ in the mat, indicating that the nitrogenase activity of the dominant cyanobacteria *Synechococcus* spp. Types A and B' is of greatest importance for this morning peak. During the night, fermentation has been proposed to contribute to the formation of H_2_ (Revsbech et al., 2016).

In this study, we identified several microbial community members that could possibly contribute to fermentative H_2_ evolution. The anaerobic fermenter, *Pseudothermotoga* sp. OTU-2, is probably the most abundant hydrogen-producing mat member. The partial genome of this bacterium contains genes encoding a [FeFe]-hydrogenase (H_2_ase; Table 3, Table S15) enabling fermentative H_2_ production, as has been shown in the next closest relative *(Pseudo)thermotoga hypogea* strain NBRC 106472 (Fardeau et al., 1997; Yang et al., 2015).

The microbial mat also contains other fermentative bacteria: e.g., an uncultured member of the *Atribacteria* (Candidate phylum OP9), OTU-7 and a member (OTU-13) of the phylum *Aminicenantes* (Candidate phylum OP8; Table 3, Table S15). Analysis of the OTU-7 metagenomic bin indicates an anaerobic, fermentative lifestyle for this mat member, similar to that deduced from single-cell genomics previously obtained for members of the phylum *Atribacteria* that occur in hot springs in California and Nevada, USA (Dodsworth et al., 2013). The presence of a gene for a monomeric, periplasmic [FeFe]-H_2_ase in the partial genome further suggests the ability of this organism to produce H_2_ fermentatively. For OTU-13 a H_2_-evolving group 4 H_2_ase (formate hydrogenlyase complex) and a bidirectional [NiFe]-H_2_ase III could be responsible for fermentative H_2_ evolution, as shown in *Thiocapsa roseopersicina* (Rákhely et al., 2007). *Thermodesulfovibrio* sp. OTU-8 may grow on fumarate and acetate, as observed for *Thermodesulfovibrio yellowstonii* (Henry et al., 1994), and may subsequently produce H_2_ via a type-IV hydrogenase (fumarate hydrogenlyase complex; Table S15).

#### H_2_ consumption

Several members of the *Chloroflexi* (OTUs-1, 11, 15), *Thermocrinis* sp. OTU-4, but also the sulfate reducing *Thermodesulfovibrio* sp. OTU-8, presumably the *Planctomycetes* member OTU-14 as well as a less abundant member of the *Thermodesulfobacteria*, OTU-26, were identified as putative H_2_ consumers due to the presence of genes encoding [NiFe]-H_2_ases (Table S15). Because it is the most abundant member of the undermat, *Roseiflexus* sp. OTU-1 is expected to be the major consumer of H_2_. The genome of *Roseiflexus* sp. strain RS-1 contains a type-I uptake [NiFe]-H_2_ase, encoded by genes *hydAB* (*hyaAB*), as well as a complete suite of *hyp* genes potentially involved in the biosynthesis and maturation of this H_2_ase. Accordingly, this organism has been suggested to utilize H_2_ (van der Meer et al., 2010). All of these genes were also present in the undermat metagenome. A high diversity (between 1 and 14 sequences) of different (partial) H_2_ases with 66–100% amino acid similarity to the *Roseiflexus* sp. RS-1 reflects the high microdiversity for this taxon (Ferris and Ward, 1997; Thiel et al., 2016) and might indicate the presence of putative ecotypes with different H_2_ases gene sequences. [NiFe]-H_2_ase genes (*hydABCD*, RoseRS_2319–RoseRS_2322) were shown to be expressed, and the relative transcript abundances for these genes exhibited a nocturnal expression pattern during the evening and night hours in the upper green layer of the microbial mat. This pattern led to the suggestion that H_2_ is an important source of electrons for hypothesized light-driven CO_2_ fixation by filamentous anoxygenic phototrophs (FAPs) in the morning, when light is available but while the mat remains anoxic (van der Meer et al., 2005; Klatt et al., 2013b).

*Thermocrinis* sp. OTU-4 is likely to be the second most abundant H_2_ consumer in the mat. Although no H_2_ase genes affiliated with this organism were detected in the assembled metagenome, their presence was confirmed in the unbinned portion of the sequences (Table S15). The closest relative of the organism(s) represented in this bin, *Thermocrinis ruber*, which was isolated from Octopus Spring from temperatures above 80°C, grows on H_2_, elemental sulfur, and thiosulfate (Huber et al., 1998; Jahnke et al., 2001; Härtig et al., 2014) and a similar metabolism can be assumed for the undermat member at 60°C based on the corresponding metagenomic bin (Table 3). The presence of affiliated H_2_ase genes only in the unassembled part of the metagenome, as well as the low number of scaffolds in the metagenome bin are indicative of assembly difficulties probably arising from the presence of closely related populations with highly similar but non-identical genome sequences. Another mat member, *Thermodesulfovibrio* sp. OTU-8, is assumed to grow on H_2_ plus acetate, as shown for its closest relative, *T. yellowstonii* (Henry et al., 1994). This suggestion is supported by the presence of genes encoding a [FeFe]-H_2_ase in the genome of the type strain and the metagenomic bin. However, genes for additional group-4 H_2_ases (formate hydrogen lyase) are present in both the isolate genome (Lim et al., 2010; Bhatnagar et al., 2015) and metagenomic bin-8 (Table S15). These genes indicate an ability to produce H_2_, e.g., when growing on formate and acetate as described by Henry et al. (1994). A less abundant putatively H_2_-utilizing member of the mat is the *Thermodesulfobacteria* member OTU-26. Genes encoding a [NiFe]-H_2_ase and an [NiFe]-H_2_ase maturation protease, closely related to sequences from *Caldimicrobium thiodismutans* have been identified in the undermat metagenome (scaffold JGI24185J35167_1006546). Additionally, H_2_ase maturation genes indicating the presence of [NiFe]-H_2_ases with unknown function and identity were identified in the novel chlorophototrophic FAP “*Ca*. R. gracile” OTU-6 and the chemotrophic *Chloroflexi* member OTU-9, as well as the unidentified *Planctomycetes* member OTU-14 (Table S15).

## Conclusion

In this study, we used deep metagenomic sequencing to further our understanding of the microbial mat community ecology at Mushroom Spring in Yellowstone National Park, and added metabolic information about the understudied orange-colored undermat layer. Genomic analyses for the 15 most abundant members of the undermat were conducted based on reference genomes and partial genomes obtained by binning a 232-Mb assembled metagenome from a full lane HiSeq sequencing run. The “cyanobacterial” mat harbors a panoply of phototrophs, some of which were initially detected in the undermat for the first time. We detected 10 of the 16 chlorophototrophs previously obtained from these mats in the undermat, and 5 of them were among the 15 most abundant members (Thiel et al., 2016; Tank et al., 2017). Although, most chlorophototrophic members were more abundant in the upper green layer, some chlorophototrophs were almost exclusively detected in the undermat (Thiel et al., 2016). The undermat portion of the community was more uneven than the upper layer. The most dominant member of the undermat is *Roseiflexus* sp., a, filamentous anoxygenic phototrophic bacterium (Thiel et al., 2016). The detection of rhodopsin genes, mostly xanthorhodopsins, in many taxa, specifically in the undermat, was surprising and strengthens the conclusion that light energy is of major importance in this community, even under very low light conditions.

The mixture of phototrophic and chemotrophic organisms in the undermat use bicarbonate as well as organic carbon sources derived from different cell components and fermentation products. The overall mat community has been suggested to ultimately depend upon the primary productivity of cyanobacteria in the upper green layer (Tank et al., 2017), which also provide the majority of nutrient and carbon sources utilized for heterotrophic and mixotrophic growth in the undermat. Further, this study indicates autotrophic growth for several undermat members. The four known bacterial carbon fixation pathways are apparently used for inorganic carbon fixation in the undermat metagenome. Additionally, inorganic carbon is probably used in anaplerotic CO_2_ fixation reactions by several undermat members of the community using 2-oxoglutarate or pyruvate:ferredoxin oxidoreductases in the reductive TCA branch as has been shown for *Cab. thermophilum* (Tank and Bryant, 2015a,b) and as hypothesized for “*Ca*. Thermochlorobacter aerophilum” (Liu et al., 2012). Nitrogen metabolism seems to be limited to assimilatory reactions. Many community members are capable of using organic nitrogen in form of proteins and amino acids. Nitrogen input into the system is likely to be driven by a low diversity of nitrogen fixing bacteria; the dominant cyanobacteria, *Synechococcus* spp. Types A and B' in the upper green layer (Steunou et al., 2006, 2008) and the anaerobe *Thermodesulfovibrio* sp. in the undermat. A closed sulfur cycle is indicated by biological sulfate reduction combined with evidence of genes for sulfide oxidation mainly in chlorophototrophs. Finally, hydrogen is produced and consumed in the mat by a variety of microorganisms, which leads to the observed diel hydrogen concentration patterns. Nitrogenase activity of cyanobacterial members of the upper layer in combination with fermentative bacteria in the undermat are the main sources of hydrogen, which can be consumed by phototrophic and chemotrophic members of the microbial mat community in both layers.

In this metagenomic analysis of the orange-colored undermat, we describe metabolic potentials and putative interactions among mat community members, leading to an initial overview of the metabolic potential of the entire mat community. Metabolic co-dependencies among various community members of both layers are indicated by (almost) closed nutrient cycles as well as nutrient, e.g., vitamin, requirements in different mat members (e.g., Tank and Bryant, 2015a,b; Tank et al., 2017). Oxygen production by the dominant upper layer cyanobacteria in concert with heterotrophic oxygen respiration in the undermat leads to a variety of oxygen microenvironments over the diel cycle affecting the microbial community and vertical distribution of different members within the mat. The analysis further disclosed the metabolic potentials of previously unknown and unidentified microbes, such as a sulfate-reducing member of the deep-branching *Bacteroidetes-Chlorobi* group, a member of the *Planctomycetes* with two types of CO_2_ fixation capabilities, and a member of the *Chloroflexi* using the Wood-Ljungdahl pathway. Although microbial studies at Mushroom Spring extend over 50 years, these microbial mats still harbor the potential for new discoveries and for gaining a deeper understanding of hot spring microbial mat ecology and physiology.

## Author contributions

VT conducted sequence analysis after assembly for metagenome sequences, including binning, phylogenetic analysis, annotation and phylogenetic marker genes analysis of metagenome bins, as well as reference targeted mapping studies, and generated tables and figures. MH analyzed carbon fixation pathway affiliated genes in the metagenome and the (partial) genomes, designed the corresponding figures, and wrote the corresponding sections of the manuscript. Sequencing, quality check, assembly, and annotation of the metagenome was conducted by JGI staff. DW and DB planned the experiments, acquired funding, organized and led field excursions, and provided scientific infrastructure. VT and DB wrote the manuscript with contributions from all other authors.

### Conflict of interest statement

The authors declare that the research was conducted in the absence of any commercial or financial relationships that could be construed as a potential conflict of interest.

## Supplementary material

Figure S1 **Schematic map of metabolic reactions of the 3-hydroxypropionate bi-cycle and enzymes involved**.

Figure S2 **Genes for rTCA cycle enzymes identified in the metagenome and affiliated with ***Thermocrinis*** sp. OTU-04**.

Figure S3 **Schematic presentation of the different versions of genes encoding an ATP dependent citrate lyase (CCS, CCL, and ACL) of the reductive TCA cycle**. Representative organisms for each gene variant are given in blue.

Figure S4 **Schematic presentation of the two branches (methyl branch and carbonyl branch) of the reductive acetyl-CoA pathway**. The two key-enzymes CO dehyodrogenase (CODH) and acetyl-CoA synthase (ACS) are given in red.

Table S1 **Reference genomes used in emergent self-organizing map binning**.

Table S2 **List of photosynthetic reaction center genes detected in the undermat metagenome, and their taxonomic affiliation**.

Table S3 **List of rhodopsin encoding genes identified in the undermat metagenome, and their taxonomic affiliation**.

Table S4 **List of bacteriorhodopsin-related protein (Brp) and bacteriorhodopsin-related protein-like homolog protein (Blh) encoding genes identified in the undermat metagenome, and their taxonomic affiliation**.

Table S5 **List of RubisCO genes detected in the undermat metagenome, length, and sequence coverage of scaffolds as well as the BLASTp results and taxonomic affiliation**.

Table S6 **List of 3-hydroxypropionate bi-cycle key-enzyme genes in metagenome bins and reference genomes of microbial mat ***Chloroflexi*****.

Table S7 **List of malonyl-CoA reductase encoding genes identified in the undermat metagenome, and their taxonomic affiliation**.

Table S8 **List of Wood-Ljungdahl/reductive acetyl-CoA pathway enzyme genes in metagenome bins and reference genomes of microbial mat members**.

Table S9 **List of genes affiliated with lactate utilization and production found in the (partial) genomes and metagenomic dataset**.

Table S10 **List of genes involved in putative utliziation of proteins and amino acids by the 15 most abundant mat members based on (partial) genome/metagenomic sequence information**.

Table S11 **List of genes affiliated with ribose utilization in (partial) genomes of the 15 most abundant mat members**.

Table S12 **List of genes assigned to nitrogen metabolism pathways in the most abundant mat member partial genomes/reference genomes and/or metagenome dataset**.

Table S13 **List of dissimilatory sulfite-reductase encoding genes (***dsrAB***) identified in the undermat metagenome, and their taxonomic affiliation**.

Table S14 **List of genes assigned to sulfur metabolism pathways in the most abundant mat member partial genomes/reference genomes and/or metagenome dataset**.

Table S15 **List of hydrogenase genes identified in (partial) genomes, reference genomes, and metagenome affiliated with the most abundant mat members**.
