# Supplementary figures and images for: The Dark Side of the Mushroom Spring Microbial Mat: Life in the Shadow of Chlorophototrophs. II. Metabolic Functions of Abundant Community Members Predicted from Metagenomic Analyses
Source: Front Microbiol. 2017 Jun 6;8:943. doi: 10.3389/fmicb.2017.00943 (PMC5459899; doi:10.3389/fmicb.2017.00943)

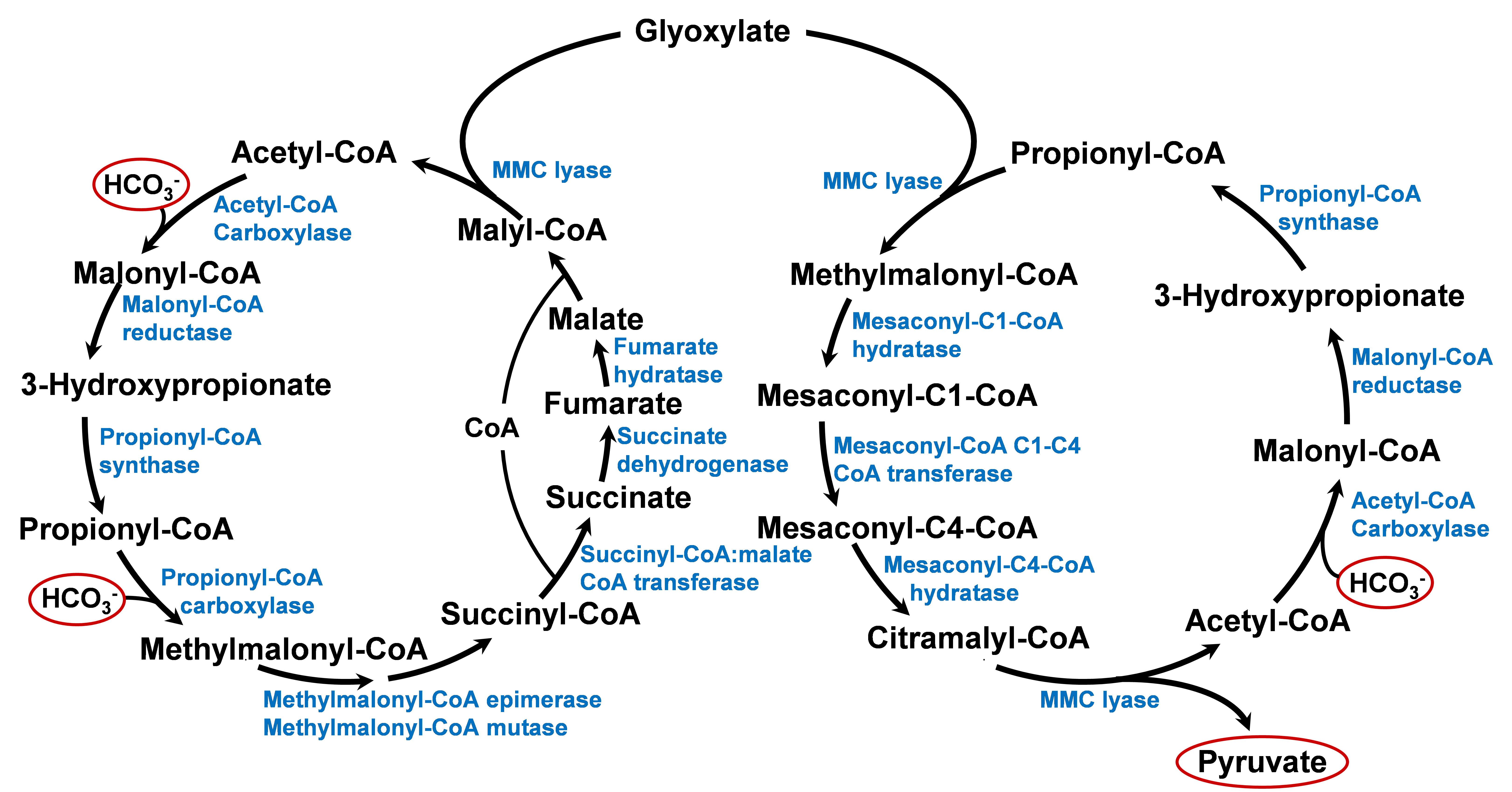

Supplement: Figure S1 — Schematic map of metabolic reactions of the 3-hydroxypropionate bi-cycle and enzymes involved. [file Image1.tif]

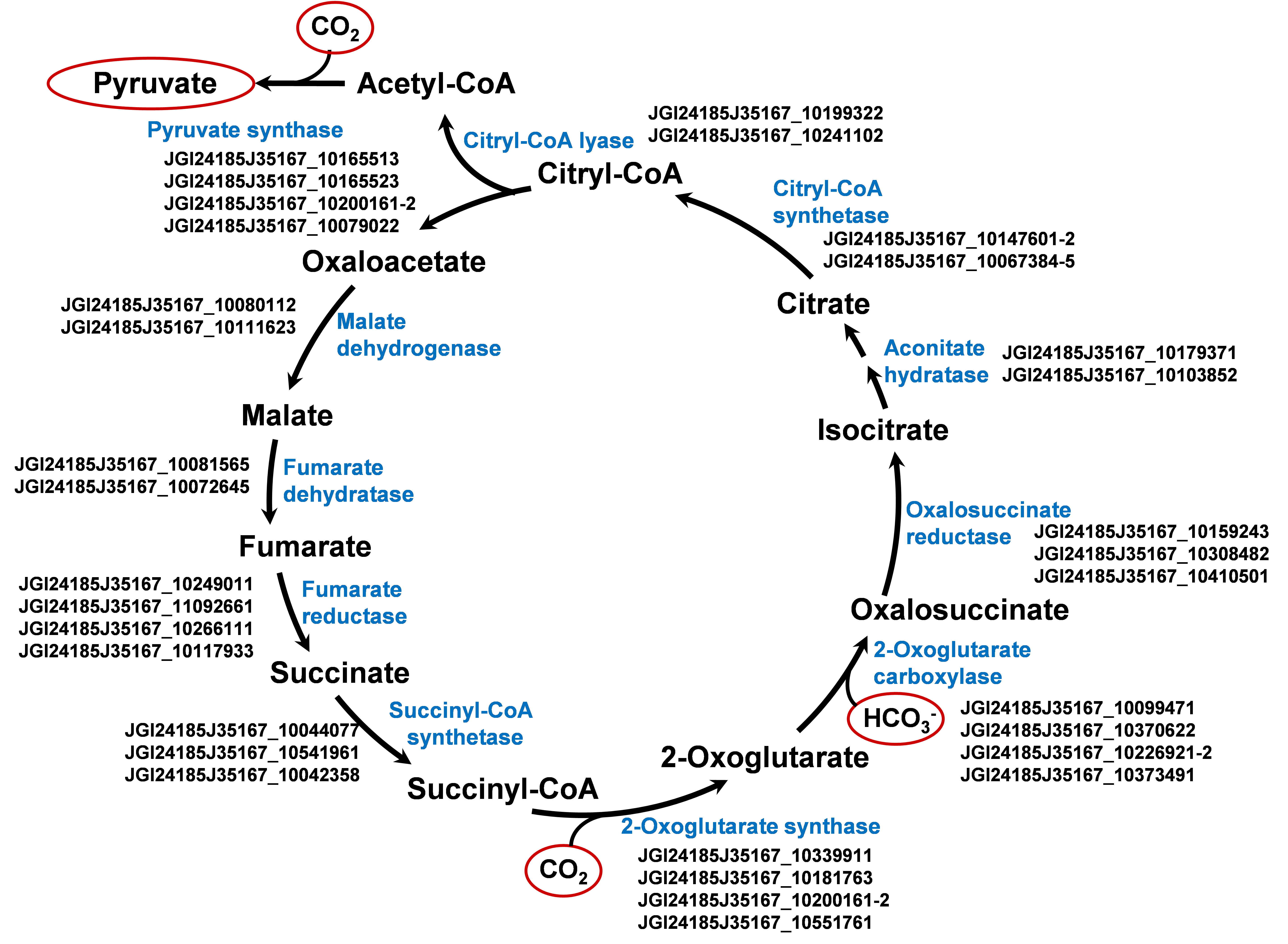

Supplement: Figure S2 — Genes for rTCA cycle enzymes identified in the metagenome and affiliated with Thermocrinis sp. OTU-04. [file Image2.tif]

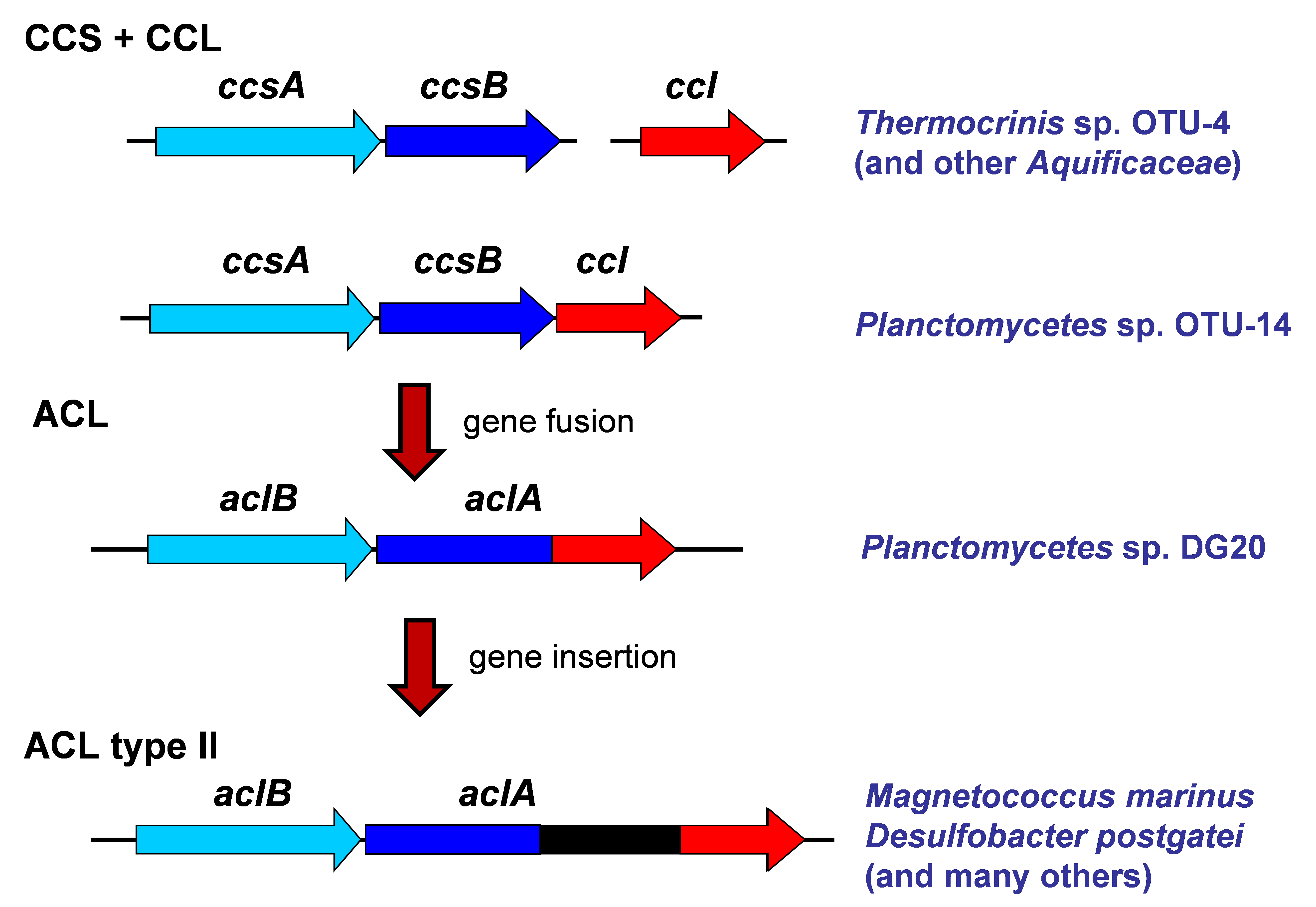

Supplement: Figure S3 — Schematic presentation of the different versions of genes encoding an ATP dependent citrate lyase (CCS, CCL, and ACL) of the reductive TCA cycle. Representative organisms for each gene variant are given in blue. [file Image3.tif]

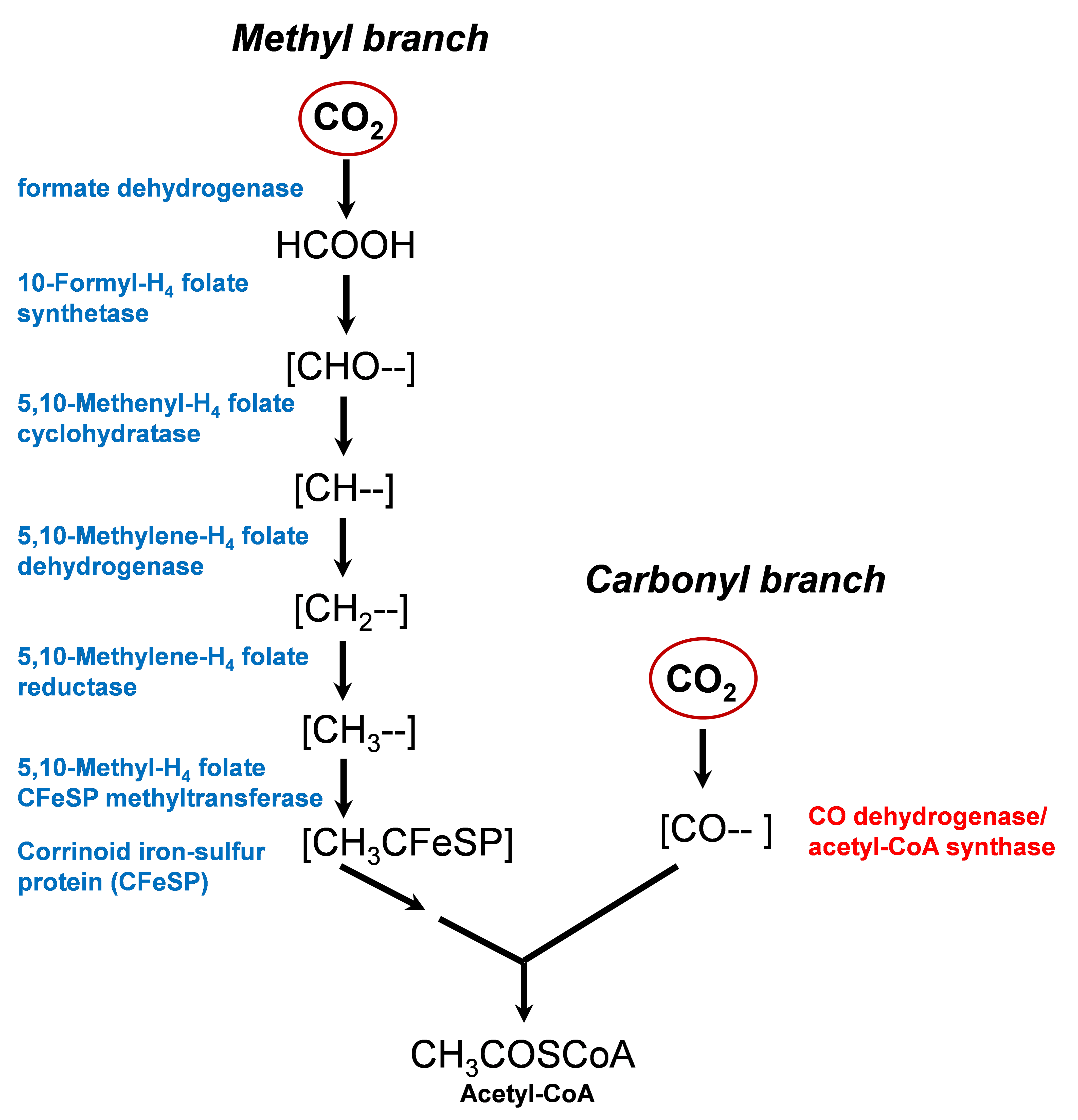

Supplement: Figure S4 — Schematic presentation of the two branches (methyl branch and carbonyl branch) of the reductive acetyl-CoA pathway. The two key-enzymes CO dehyodrogenase (CODH) and acetyl-CoA synthase (ACS) are given in red. [file Image4.tif]
